# SOX4 maintains the stemness of cancer cells via transcriptionally enhancing HDAC1 revealed by comparative proteomics study

**DOI:** 10.1186/s13578-021-00539-y

**Published:** 2021-01-22

**Authors:** Jingshu Liu, Jiangfeng Qiu, Zhiqi Zhang, Lei Zhou, Yunzhe Li, Dongyan Ding, Yang Zhang, Dongling Zou, Dong Wang, Qi Zhou, Tingyuan Lang

**Affiliations:** 1 College of Bioengineering, Chongqing University, 400044 Chongqing, People’s Republic of China; 2 Department of Gynecologic Oncology, Chongqing University Cancer Hospital, 400030 Chongqing, People’s Republic of China; 3 Chongqing Key Laboratory of Translational Research for Cancer Metastasis and Individualized Treatment, Chongqing University Cancer Hospital, 400030 Chongqing, People’s Republic of China; 4 Key Laboratory for Biorheological Science and Technology of Ministry of Education (Chongqing University), Chongqing University Cancer Hospital, 400044 Chongqing, People’s Republic of China; 5 Department of Gastrointestinal Surgery, Renji Hospital Shanghai Jiao Tong University School of Medicine, 200127 Shanghai, People’s Republic of China; 6 Department of General Surgery, School of Medicine, Shanghai Fourth People’s Hospital Affiliated to Tongji University, 200081 Shanghai, People’s Republic of China; 7 Singapore Eye Research Institute, The academia, 20 College Road, Discovery Tower Level 6, 169856 Singapore, Singapore; 8 Department of Ophthalmology, Yong Loo Lin School of Medicine, National University of Singapore, Singapore, Singapore; 9 Ophthalmology and Visual Sciences Academic Clinical Research Program, Duke-NUS Medical School, National University of Singapore, Singapore, Singapore; 10 Laboratory Department, Chongqing University Cancer Hospital, 400030 Chongqing, People’s Republic of China

**Keywords:** Cancer stem cells, Comparative proteomics, SOX4, HDAC1, Transcriptional activation, Precision medicine

## Abstract

**Background:**

Cancer stem cells (CSCs) are the root of human cancer development and the major cause of treatment failure. Aberrant elevation of SOX4, a member of SOX (SRY-related HMG-box) family transcription factors, has been identified in many types of human cancer and promotes cancer development. However, the role of SOX4 in CSCs, especially at a proteome-wide level, has remained elusive. The aim of this study is to investigate the effect of SOX4 on the stemness of CSCs and reveal the underlying mechanisms by identification of SOX4-induced proteome changes through proteomics study.

**Results:**

Overexpression of SOX4 promotes sphere formation and self-renewal of colorectal cancer cells in vitro and in vivo and elevates the expression levels of CSCs markers. Through iTRAQ-based quantitative proteomics analysis, 215 differentially expressed proteins (128 upregulated, 87 downregulated) in SOX4-overexpressing HCT-116 spheres were identified. The bioinformatic analysis highlighted the importance of HDAC1 as the fundamental roles of its impacted pathways in stem cell maintenance, including Wnt, Notch, cell cycle, and transcriptional misregulation in cancer. The mechanistic study showed that SOX4 directly binds to the promoter of HDAC1, promotes HDAC1 transcription, thereby supporting the stemness of colorectal cancer cells. HDAC1 hallmarks colorectal cancer stem cells and depletion of HDAC1 abolished the stimulatory effect of SOX4. Furthermore, SOX4-HDAC1 axis is conserved in multiple types of cancer.

**Conclusions:**

The results of this study reveal SOX4-induced proteome changes in HCT-116 spheres and demonstrates that transcriptional activation of HDAC1 is the primary mechanism underlying SOX4 maintaining CSCs. This finding suggests that HDAC1 is a potential drug target for eradicating SOX4-driven human CSCs.

## Introduction

Cancer is a highly heterogeneous malignant disease that consists of at least two types of subpopulations: a small number of self-renewing cancer stem cells (CSCs), also named tumor-initiating cells (TICs), and a majority of differentiated cancer cells [1, 2]. CSCs are the major cause of therapeutic resistance, metastasis, and recurrence, ultimately, leading to treatment failure [3]. However, despite central roles, the mechanisms underlying their regulation are incompletely understood.

SOX (SRY-related HMG-box) family proteins are a group of evolutionarily conserved transcription factors containing at least 20 members grouped into eight classes (SoxA to SoxH) that present a highly conserved high-mobility group (HMG) domain and play fundamental physiological and pathological roles, such as cell fate decision, testis determination, male fertility as well as normal organ and cancer development [4–6]. SOX4 belongs to the SoxC subgroup which is essential for human organ development [5, 6]. SOX4 also plays pivotal roles in cancer development; it aberrantly upregulates in various types of cancer, including breast cancer [7], lung cancer [8, 9] as well as cervical cancer [10] and induces epithelial-to-mesenchymal transition (EMT), by activating TGF-β pathway in breast and gastric cancers and/or by PTEN ablation in prostate cancer [11–13]. While, despite the importance of SOX4 in cancer development, its role in stemness maintenance of human cancer has not been fully understood.

HDAC1, a member of histone deacetylases (HDAC) family, is responsible for removing the acetyl groups from the ε-N-acetyl lysine amino acid on histone tails to regulate gene transcription [14]. HDAC1 is essential for cancer development: high expression of HDAC1 predicts poor prognosis in multiple cancer types, such as lung [15], breast [16, 17], ovarian [16], prostate [18], and renal [19] cancer. The mechanisms underlying HDAC1-driven cancer development involve in several cellular processes, including cell cycle, apoptosis, DNA-damage response, and autophagy [20]. In breast cancer, HDAC1 is associated with ERα expression [21]. Several HDAC1 inhibitors have been developed for anti-cancer applications [20]. While, despite massive studies, the upstream regulation of HDAC1 and its role in cancer stemness remain elusive.

Mass spectrometry-based comparative global proteomics holds promise to investigate disease mechanisms through identifying proteome changes under pathological conditions [22]. Although exhaustive studies aimed at identifying the downstream targets of SOX4 have been performed on the genome- and transcriptome-wide levels in various types of cells, such as human mammary epithelial cell line (HMLE) [23], embryonic neural stem cells [24], small cell lung cancer cells [25], acute lymphoblastic leukemia cells [26], the SOX4-induced proteome changes, especially in human CSCs, have not been investigated, which would be important for the investigation of the mechanisms underlying SOX4-driven cancer stemness and development of a novel strategy of SOX4 inhibition.

In this study, by identification of SOX4-induced proteome changes in colorectal cancer stem cells (CRC-SCs), we investigated the potential molecular effects of SOX4 in CRC-SCs and found that SOX4 transcriptionally regulates HDAC1 to support the stemness of CSCs. This work reveals a novel underlying mechanism, SOX4-HDAC1 axis, for stemness maintenance of human cancer and suggests that HDAC1 inhibition should be an effective precision therapeutic strategy for eradicating SOX4-drived human CSCs.

## Materials and methods

### Ethical issues

All the protocols involved in animals were approved by the Institutional Animal Care and Use Committee at Chongqing University Cancer Hospital and the experiments were performed in accordance with the National Institute of Health Guide for the Cancer and Use of Laboratory Animals. The clinical samples used in this study were collected from Shanghai Jiao Tong University Affiliated Sixth People’s Hospital East Campus with written informed consent. The experiments were approved by the ethics committee of Chongqing University Cancer Hospital.

### Cells and cell culture condition

The human colorectal cancer cell (CRC) lines HCT-116 and HT-29, human ovarian cancer cell lines Caov-3 and SK-OV-3, human breast cancer MCF7, MDA-MB-361 and SK-BR-3, human lung cancer A549 and NCI-H460, human gastric cancer AGS and NCI-N87, human liver cancer HEPG2 and SNU-182 cell lines were purchased from the American Type Culture Collection (ATCC). HCT-116, HT-29, SK-OV-3, and SK-BR-3 cells were cultured in McCoy’s 5A medium (Thermo Fisher Scientific). MCF7 and HEPG2 cells were cultured in Eagle’s Minimum Essential Medium (Thermo Fisher Scientific). Caov-3 cell was cultured in Dulbecco’s Modified Eagle’s Medium (Thermo Fisher Scientific). MDA-MB-361 cell was cultured in Leibovitz’s L-15 medium (Thermo Fisher Scientific). A549 and AGS cells were cultured in F-12 K medium (Thermo Fisher Scientific). NCI-H460, NCI-N87, and SNU-182 cells were cultured in RPMI-1640 medium (Thermo Fisher Scientific). The complete growth medium was produced by the addition of 1 % penicillin-streptomycin (Thermo Fisher Scientific) and 10 % fetal calf serum (Thermo Fisher Scientific). The e-Myco VALiD Mycoplasma PCR Detection Kit (iNtRon Biotechnology) was used to confirm the absence of mycoplasma. All cell lines used in this study were examined by short tandem repeat profiling.

### Primary normal colorectal cancer (CRC) cell culture

Tissue samples were collected from the primary tumor of colorectal cancer patients undergoing surgical resection and were maintained in cold PBS (Thermo Fisher Scientific) with antibiotics (500 U ml^− 1^ penicillin, 500 µg ml^− 1^ streptomycin, 100 µg ml^− 1^ gentamicin, and 2.5 µg ml^− 1^ amphotericin B) during transportation. The tissues were then removed from the fatty and necrotic areas and dissected into pieces (1 mm^3^). After digestion in RPMI-1640 medium containing 40 U ml^− 1^ collagenase, the samples were sequentially passed through a 40 µm (Corning) and a 30 µm (Miltenyi Biotec) cell strainer filter. The resulting cells were washed and cultured in complete culture medium (RPMI-1640 medium supplemented with 1 % penicillin-streptomycin (Thermo Fisher Scientific) and 10 % fetal calf serum). The cells were passaged at 70–80 % confluence.

For primary spheroid culture, the pieces were maintained in serum-free RPMI-1640 medium supplemented with 2 % B-27 supplement (Thermo Fisher Scientific), 20 ng ml^− 1^ fibroblast growth factor 2 (FGF2) (Thermo Fisher Scientific) and 20 ng ml^− 1^ epidermal growth factor (EGF) (Thermo Fisher Scientific) and the single cells were cultured in ultra-low attachment 6 well plate containing StemPro hESC SFM (Thermo Fisher Scientific) supplemented with 8 ng ml^− 1^ FGF2 and antibiotics. The spheroids were passaged at 70–80 % confluence by centrifuge.

### Sphere formation assay

The cells were dissociated into single cells by trypsinization and filter as mentioned above and 1,000 dissociated single cells were plated into an ultra-low attachment plate containing StemPro hESC SFM supplemented with 8 ng ml^− 1^ FGF2 and antibiotics. After 10 days of culture, spheres with diameters above 50 µm were counted under an inverted microscope (Leica). The forming efficiency (%) = counted sphere number/total plating cells.

For examination of sphere-forming activity on serial passages, the spheres were collected and trypsinized at 10 days post-plating, followed by single-cell production as mentioned above. The culture medium was then changed by centrifugation and 1000 single cells were replated in ultra-low attachment 6 well plate for a new round of assay.

### Limiting dilution assay

The cells were dissociated into single cells by trypsinization and filter and maintained in serum-free DMEM/F12 medium containing B27 supplement, 2 mM _L_-glutamine (Thermo Fisher Scientific), 10 ng ml^− 1^ FGF2, 20 ng ml^− 1^ EGF, 5ug ml^− 1^ insulin (Thermo Fisher Scientific) and 0.4 % bovine serum albumin (Thermo Fisher Scientific). The cells were then plated into u-bottom ultra-low 96-well cell culture plate (Corning) at the density of 10, 5, or 1 cells per well. The number of wells in which the sphere formation can be observed was scored after ten days of culture. The frequency of sphere-forming cells was calculated by ELDA online software [27]. Three replicates were performed for each group.

For in vivo LDA assay, the single cells derived from suspension-cultured spheres were suspended in a mixture of DMEM/F12 and Matrigel (BD Biosciences) (1:1) and 21, 7, 3 viable cells were injected subcutaneously into the flanks of 6 weeks old male NOD/SCID mice. Tumor formation was examined weekly for 35 days and the number of mice with tumor formation was scored. The frequency of tumor-initiating cells was calculated by ELDA online software [27]. Each group was performed in triplicate.

### Plasmids construction

For overexpression of SOX4 and HDAC1, the cDNAs were amplified by primeSTAR HS DNA polymerase (Takara) and inserted into pCDH-CMV-MCS-EF1-Puro plasmid (Addgene). For knockdown, the pLKO.1 lentivirus plasmid containing shRNAs specific against SOX4 (TRCN0000018214, TRCN0000018217) or HDAC1(TRCN0000349639, TRCN0000197176) were purchased from Sigma-Aldrich. pLKO.1-puro plasmid containing non-target shRNA (SHC016-1EA, Sigma-Aldrich) was used for control. For luciferase reporter assay, the wild type, F1, F2 and F3 fragments of HDAC1 promoter were amplified by polymerase chain reaction (PCR) from the genomic DNA of HCT-116 cells and subcloned into the pGL-4.23 plasmid (Promega). The blank mt, mt1, mt2, mt3 and mt4 HDAC1 promoters were obtained by PCR amplification of synthesized corresponding DNA fragments. phRL-TK (Promega) Renilla luciferase reporter plasmid was used as an internal control. All reconstructed plasmids were confirmed by sequencing. The sequences of primers used were listed in Additional file 1: Table S1.

### Lentivirus production and cell infection

HEK293T (Clontech) cells were transfected with reconstructed lentivirus plasmids together with packaging plasmid (pCMV-dR8.91) and envelope plasmid (pCMV-VSV-G) at a ratio of 5:4:1 using jetPEI (PolyPlus Transfection). The medium was changed at 24 h after transfection. After another 24 h culture, the virus was collected, filtered (0.45 µm), and concentrated (24,000 rpm, 2 h). Medium containing virus and 8 µM ml^− 1^ polybrene (Thermo Fisher Scientific) was used for cell infection. The infected stable cells were selected by medium containing 1 µg ml^− 1^ puromycin for 1 week. The performance of genetic manipulation was examined by western blot.

### Real‐time quantitative reverse transcription PCR (real‐time qRT-PCR)

Total RNA was extracted using a RNeasy Mini Kit (Qiagen). cDNA was synthesized by an iScript cDNA synthesis kit (Bio-Rad). Real-time qRT-PCR was performed in a reaction mixture of SYBR-green by StepOne Real-Time PCR System (Thermo Fisher Scientific). The expression of genes of interest was normalized to the internal control (human GAPDH) and calculated by the comparative threshold cycle (*Ct*) method. The sequences of primers used in this study were listed in Additional file 1: Table S1.

### Western blot

The cells were cultured in 75 mm cell culture dish (Corning). After reaching 80 % confluence, the cells were washed by ice-cold PBS at least three times. 1 ml Radioimmunoprecipitation assay buffer (RIPA, Thermo Fisher Scientific) supplemented with protease inhibitor cocktail (Roche) was then added to the cells and kept on ice for 5 min. The lysate was then collected and transferred to a microcentrifuge tube. After centrifugation (14,000 × *g*, 15 min), the supernatant was collected for further analysis. The total protein samples were boiled with SDS-PAGE loading buffer (Bio-Rad) for 5 min followed by SDS-PAGE separation. The samples were then transferred to polyvinylidene difluoride membranes (Thermo Fisher Scientific) according to the standard protocol. The membranes were blocked with bovine serum albumin or nonfat milk and sequentially incubated with first antibodies and HRP-conjugated secondary antibodies. The signal was achieved by incubating the membranes with ECL Western Blotting Substrate (Thermo Fisher Scientific). Signals were visualized with an imaging system (Bio-Rad). Antibodies used in this study were listed in Additional file 1: Table S2.

### Flow cytometry

The gene-manipulated cells and control cells were lysed by trypsin and washed three times with PBS containing 10 % fetal calf serum, and then incubated with fluorescein isothiocyanate-conjugated rabbit anti-human CD133 or anti-human CD44 antibodies in PBS containing 10 % fetal calf serum. Rabbit IgG was used as isotype control. After incubation, the cells were washed three times with PBS containing 10 % fetal calf serum, and then analyzed by flow cytometry. Antibodies used in this study were listed in Additional file 1: Table S2.

### iTRAQ quantitative analysis

**Fig. 1:**
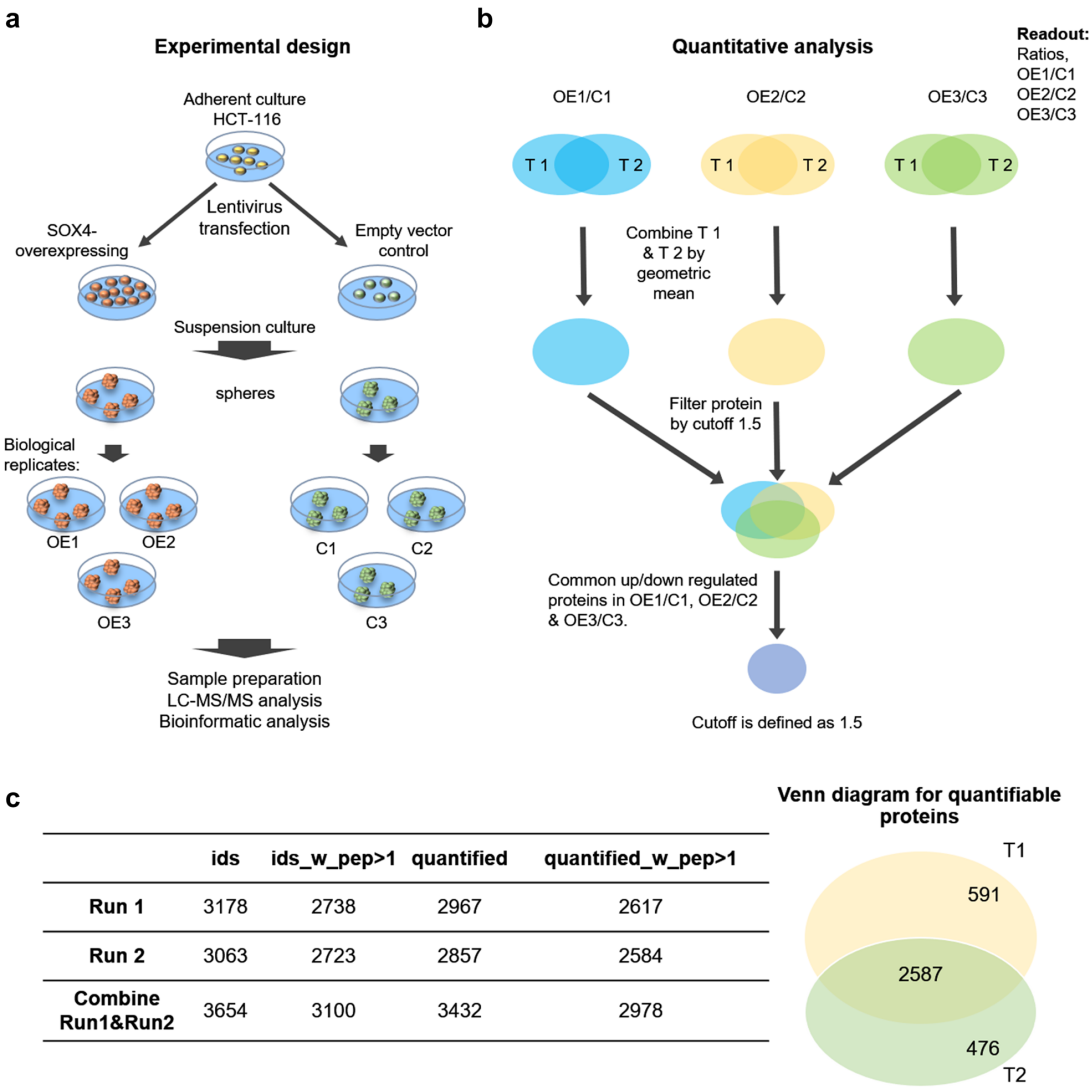
Proteomic analysis of HCT-116 cells with and without SOX4 overexpression. **a** Experimental design of the proteomic analysis. HCT-116 cells were infected with virus-containing SOX4-overexpressing vectors Overexpression group (OE) or empty vectors Control group (C). The selected stable cells were then suspension-cultured for sphere formation. Three biological repeats were performed. Proteins isolated from spheres were subjected to nanoLC-RP-MS/MS analysis. **b** Workflow of data analysis. Three biological repeats (blue, yellow, and green) and two technical repeats (T1 and T2) were performed. The data that was observed in each group and all three biological repeats and the coefficient of variation was less than 30 % between two technical repeats were kept for further analysis. **c** The results of nanoLC-RP-MS/MS analysis. In total, 3654 unique proteins were identified with high confidence (false discovery rate < 1 %) in which 3100 proteins were identified with at least two peptide fragments. Among them, 3432 proteins were quantifiable in which 2978 proteins were quantifiable with more than two peptide fragments. 2587 out of 3654 proteins were seen in two technical runs indicating the highly reproducible results of this study

The experiment design of the proteomics study is presented in Fig. 1a. Notably, the spheres were used. iTRAQ analysis was performed as described previously [28, 29] with minor modified. Briefly, SOX4-overexpressing HCT-116 and control cells were cultured in ultra-low attachment plate for sphere formation. Three biological repeats were performed. The spheres were collected by centrifugation and 1 × 10^7^ digested cells were lysed by a mixture of acetonitrile and 50 mM ammonium bicarbonate (1:9) containing 0.1 % (w/v) ProteaseMax powder (Promega). The lysis was then centrifuged (14,000 rpm, 7 min, room temperature) and the concentration of the total protein in the supernatant was measured. An aliquot of 100 µg protein of each group (3 controls and 3 SOX4-overexpressing cell samples) was used for iTRAQ 6-plex analysis (6 arms used in iTRAQ-8 plex reagent) as shown in Fig. 1b. The protein samples were incubated with 50 mM TCEP for 1 h at 60 °C and transferred to a centrifuge tube with a 30 kDa cut-off filter membrane inside and subsequent alkylation and digestion were performed in the membrane. The samples in the membrane were first incubated with 75 % urea solution followed by centrifugation to remove SDS. The samples were then incubated with IAA (15 mM) for 30 min at room temperature in dark for alkylation. After 3 times washes with 75 % urea solution, the samples were incubated with 0.1 M Triethylammonium bicarbonate solution (TEAB). After digestion with trypsin (1:50) for 16 h at 37 °C, the peptides were eluted with a mixture of 0.1 M TEAB and 0.5 M sodium chloride. The peptides were dried and incubated with iTRAQ labeling reagents for 2 h at room temperature. All labeled samples were then pooled for subsequent fractionation.

Waters HPLC system (Elstree, UK) was used for high pH reverse phase (RP) fractionation. Briefly, the samples were resolved in mobile phase A and injected into the system. The conditions were as follow: column - Waters Acquity UPLC BEH C18, 3.5 µm, 3.0 × 150 mm; mobile phase A − 20 mM ammonium formate (pH 10); mobile phase B − 80 % Acetonitrile in 20 mM Ammonium formate (pH 10); flow − 0.4 mL/min; gradient − 5–15 % in 20 min, 15–40 % in 20 min, 40–80 % in 1 min. A total of 10 fractions were collected for further nano-LC-MS/MS analysis.

NanoLC-MS/MS analysis was performed by Ultimate 3000 nanoLC system (Thermo Fisher Scientific) coupled with AB Sciex 5600 TripleTOF (AB Sciex). Two technical replicates were performed for each fraction. The conditions were as follow: column - Acclaim PepMap RSLC C18, 15 cm × 75 µm; mobile phase A − 0.1 % formic acid, 2 % acetonitrile in water; mobile phase B- 2/98 v/v of water/ACN with 0.1 % formic acid; flow – 3.00 nL/min; gradient − 5–12 % in 30 min, 12–40 % in 60 min, and 40–90 % in 20 min.

The parameters for the mass spectrometer were as follows: ISVF (Ionspray Voltage Floating) = 2000 V, CUR (curtain gas) = 30, GS2 (Ion source gas 1) = 10, IHT (Interface Heater Temperature) = 125, DP (declustering potential) = 100 V, NC (Nebuliser current) = 3 for nitrogen gas. IDA (information-dependent acquisition) mode and Analyst TF 1.7 software (AB Sciex) was used for data acquisition. The settings for IDA were as follows: time-of-flight mass spectrometry survey scan − 0.25 s; mass range − 400–1250; followed by product ion scan − 0.05 s; mass range: 100–1500. The settings of Switching criteria were as follows: mass range = 400 to 1250, charge state = 2 ~ 5; maximum number of candidate ions to monitor per cycle = 40 spectra; abundance threshold > 120 counts. 12 s was used for the exclusion of former target ions. IDA Advanced settings such as ‘dynamic accumulation’, ‘adjust CE when using iTRAQ Reagent’ and ‘rolling collision energy (CE)’ was included.

Data analysis was performed by employing ProteinPilot 5.0 software (AB SCIEX) and the protein database (version: uniprot_all_Oct2014) base on typical settings provided previously. Protein identification was performed by ProteinPilot software. Relative quantitation was performed with Pro Group algorithm in ProteinPilot base on peak areas of reporter ions. The analysis strategy considering biological and technical replicates shown in Fig. 1a–c.

### Bioinformatics analysis

Gene ontology (GO) and Pathway analysis was performed with iPathwayGuide online tool [30]. The protein-protein interaction (PPI) network was constructed based on STRING database [31] and visualized and analyzed in Cytoscape [32]. The PPI network modules were further identified with Cytoscape plugin algorithm – Molecular Complex Detection (MCODE) [33]. Module identification criteria included degree cutoff of 2, node score cutoff of 0.2, k-core of 2, haircut (on), fluffing (off), and maximum depth of 100. Significant modules were identified with MCODE score > 5.

#### Magnetic‐activated cell sorting (MACS)

HCT-116, HT-29, and primary colorectal cancer cells positive for CD44 marker were isolated by MACS (Miltenyi Biotec) as described by Jue Wang’s publication [34]. Briefly, CD44 + cells were isolated by incubation of 1 × 10^7^ cells with CD44 MicroBeads (Miltenyi Biotec) for 15 min at 4 °C in the dark followed by washing and magnetic sorting.

### Luciferase reporter assay

The HDAC1 promoter containing pGL-4.23 plasmids and phRL-TK plasminds were transiently co-transfected into indicated cells by lipofectamine 2000 (Thermo Fisher Scientifc). After 24 h transfection, the luciferase activity was measured using the Dual-luciferase Reporter Assay system (Promega). The luciferase signal was normalized by *Renilla reniformis* luciferase signal. Three replicates were performed for each group.

### Chromatin immunoprecipitation (ChIP) assay

MAGnify™ Chromatin IP System (Thermo Fisher Scientific) was used for ChIP assay. Briefly, the cells were normally cultured until 80 % confluence, followed by crosslink with 1 % formaldehyde at room temperature for 10 min. The reaction was quenched by 0.125 M glycine for 5 min. The cells were then collected by a scraper and transferred to a microcentrifuge tube. The cells were washed with cold PBS at least three times through centrifugation (200 × g, 10 min) at 4 °C, followed by lysis with a lysis buffer supplemented with proteinase inhibitor for 1 h at 4 °C. To produce 200–500 base pair DNA fragments, the lysis was sonicated on ice. After 10 min centrifugation (20,000 × g), the supernatant containing chromatin was collected and transferred to a new tube. Chromatin samples were diluted in 100 µl ice-cold dilution buffer supplemented with complete protease inhibitors cocktail and Dynabeads protein A/G were prepared in cold dilution buffer containing SOX4 antibody. The chromatin was incubated with SOX4 antibody-Dynabeads protein A/G complex for at least 18 hours at 4 °C. The beads were sequentially washed with IP buffer 1 and IP buffer 2 at 4 °C five times. Beads were then separated and incubated in a cross-linking buffer containing proteinase K at 55 °C for 15 min followed by another incubation in a new sterile tube for 30 min at 65 °C. DNA samples were isolated by incubation with DNA purification magnetic beads in DNA purification buffer for 5 min at room temperature followed by washing with DNA wash buffer and extraction with DNA elution buffer sequentially. The purified DNA was used for further quantification of DNA of interest immunoprecipitated with SOX4 protein. The primers used were listed in Additional file 1: Table S1.

### DNA pull‐down assay

The biotin-labeled HDAC1 promoters were prepared with Biotin 3’ End DNA labeling Kit (89,818, Thermo Fisher Scientific) according to the manual. Briefly, 5 pmol DNA samples were incubated with the TdT (Terminal Deoxynucleotidyl Transferase) reaction buffer containing 0.5 µM biotin-11-UTP and 0.15 U/µl TdT at 37 °C for 30 min. The reaction was stopped by 0.2 M EDTA. To remove the TdT, 50 µl of chloroform:isoamyl alcohol was added, followed by centrifugation. Then, the aqueous phase was collected. To immobilize DNA, the Dynabeads™ M-270 Streptavidin (65,305, Thermo Fisher Scientific) was used according to the manual. Briefly, the beads were resuspended in B&W buffer (binding and washing buffer) (5 µg/µl) and equal volume of biotinylated DNA was added, followed by incubation (15 min). The DNA coated beads were separated by a magnet. After 3 times washing with B&W buffer, the beads were used for downstream application. Nuclear extracts from 2 × 10^7^ cells were subsequently prepared and incubated with poly(deoxyinosinic-deoxycytidylic) acid in binding buffer (20 mM HEPES-KOH (pH 7.9), 100 mM NaCl, 1.5 mM MgCl_2_, 0.1 % (v/v) Nonidet P-40 and protease inhibitors) at a 250:1 (w/w) ratio (room temperature, 10 min). The DNA coated beads were then added and incubated at 25 °C for 30 min. After washing three times with binding buffer, the bead-bound DNA-protein complexes were eluted with Tris buffer (pH 6.8) containing 2 % SDS, 0.3 M β-mecaptoethanol, 0.05 % bromphenol blue, and 10 % (v/v) glycerol, (0.05 %) (95 °C, 5 min). The protein samples eluted from the beads were subsequently subjected to western blot assay for SOX4 detection.

### Data analysis

Data were represented as ‘mean ± SD’ from three experiments except where indicated. The significance was determined by Student’s *t*-test (unpaired, two-tailed, *P < 0.05, **P < 0.01, ***P < 0.001). Kaplan-Meier analysis was used to compare colorectal cancer patient survival based on HDAC1 expression.

## Results

### SOX4 promotes the stemness of CRC cells

**Fig. 2:**
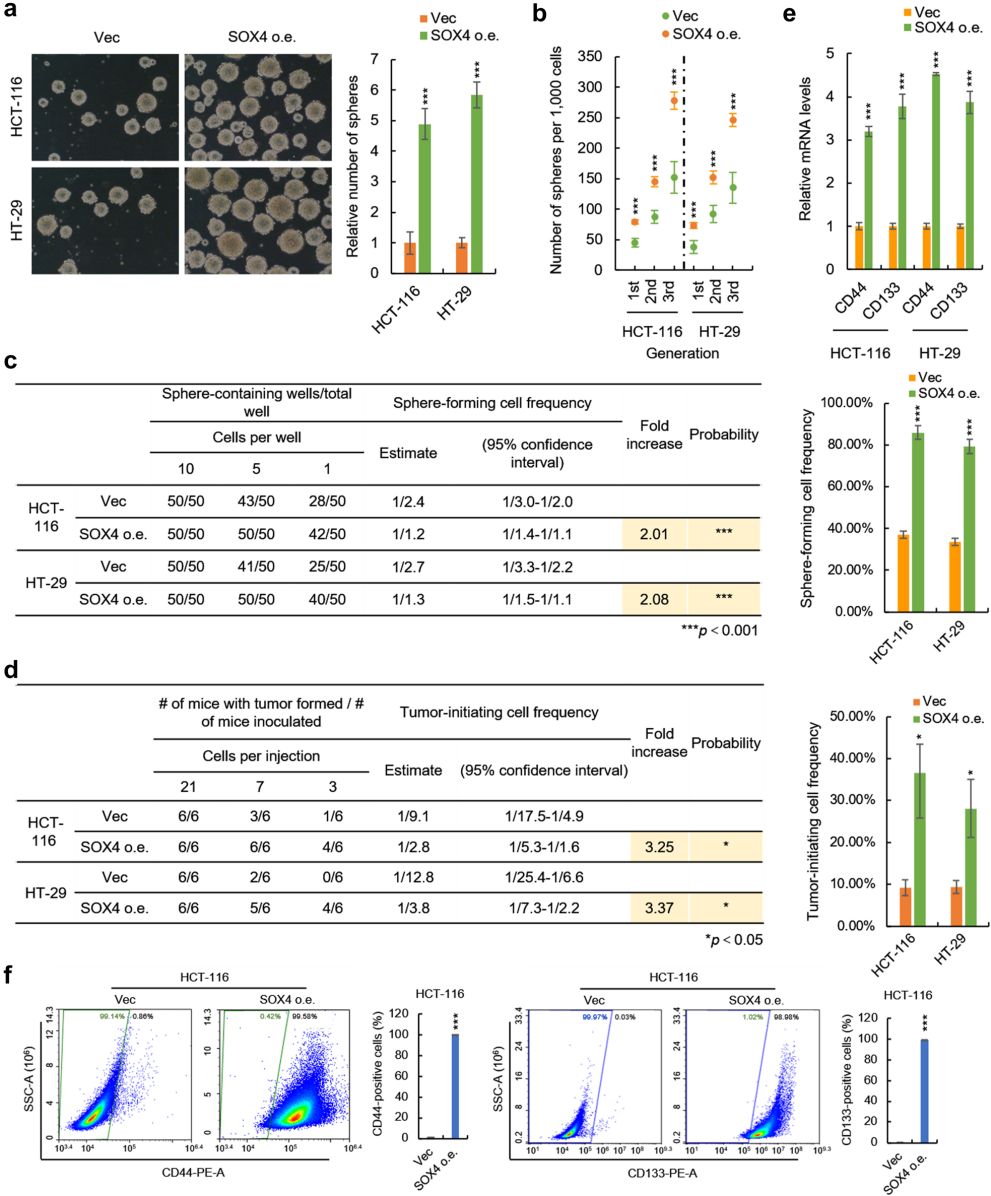
SOX4 promotes the stemness of colorectal cancer cells. **a** SOX4 promotes the sphere-forming capacity of colorectal cancer cells. The SOX4-overexpressing and control cells were seeded in ultra-low attachment 6 well plate. The number of spheres was counted after 10 days. **b** SOX4 promotes the self-renewal capacity of colorectal cancer cells on serial passage. The sphere number of indicated primary, secondary, and tertiary passaged cells was counted after 10 days. **c** SOX4 promotes sphere-forming cell frequency of colorectal cancer cells. SOX4-overexpressing and control cells were seeded into 96-well U-bottomed culture plates at a density of 10, 5 or 1 cells per well and cultured for 10 days. The sphere-forming cell frequency was calculated by ELDA software. **d** SOX4 promotes tumor-initiating cell frequency of colorectal cancer cells. SOX4-overexpressing and control cells were injected into the subcutaneous tissues of 6-week-old nude mice at a density of 21, 7, 3 cells per mouse. The number of mice developed tumors was counted after 35 days. The tumor-initiating cell frequency was calculated by ELDA software. **e**, **f** SOX4 promotes the expression of colorectal cancer stem cell markers in colorectal cancer cells. **e** The mRNA levels of CD44 and CD133 in SOX4-overexpressing and control HCT-116 and HT-29 cells were analyzed by qRT-PCR. **f** The surface protein levels of CD44 and CD133 in SOX-4 expressing and control HCT-116 cells were analyzed by flow cytometry. Data are represented as mean ± s.d.; Data are represented as mean ± s.d.; *P<0.05, **P<0.01, ***P<0.001; two-tailed Student’s *t*-test

To determine the role of SOX4 in stemness maintenance of human CSCs, we established SOX4-overexpressing CRC cells (HCT-116 and HT-29) by lentivirus delivery system (Additional file 1: Fig. S1a) and the stem cell characteristics were examined. Sphere-formation in floating culture condition is one of the most predominant characteristics of CSCs which is usually employed to assess cell stemness [35]. By sphere-formation assay, we found that SOX4 significantly enhanced the sphere-forming capacity of both HCT-116 and HT-29 cells reflected by the number of the spheres (Fig. 2a).

CRCs possess the ability to maintain the undifferentiated state over passages, termed self-renewal [1, 2]. We next investigated the effect of SOX4 on the self-renewal capacity by examination of the number of passaged spheres derived from SOX4-overexpressing cells and their control cells. As shown in Fig. 2b, SOX4 increased the number of all primary, secondary, and tertiary passaged spheres, indicating that SOX4 promotes self-renewal of CRC cells.

To further confirm the stimulatory effect of SOX4 on the stemness of CRC cells, we examine the frequency of sphere-forming and tumor-initiating cells of SOX4-overexpressing cells and their control cells by LDA assay. As shown in Fig. 2c, d and Additional file 1: Fig. S1b, c, the results from LDA assay showed that the frequencies of both in vitro sphere-forming cells and in vivo tumor-initiating cells were significantly increased by SOX4 overexpression, which confirms that SOX4 enhances the sphere-forming and self-renewal capacities of CRC cells.

Furthermore, CD44 and CD133 are well-identified surface markers of CRC-SCs [36], we found that overexpression of SOX4 significantly increased the expression of CD44 and CD133 in HCT-116 and HT-29 cells revealed by qRT-PCR (Fig. 2e). This result was further confirmed by flowcytometry in HCT-116 cell (Fig. 2f). Taken together, the above results demonstrate that SOX4 promotes the stemness of CRC cells.

### Proteomics analysis of SOX4-overexpressing CRC cells

Proteomics analysis is a robust tool for dissecting downstream signaling of a gene at the proteome level. We next employed iTRAQ-based quantitative proteomics technology to investigate the proteome changes induced by SOX4 overexpression. The experiment design and workflow are presented in Fig. 1a, b. The SOX4-overexpressing HCT-116 cells and empty vector control cells were first suspension-cultured for sphere-formation, followed by total protein extraction and proteomics analysis. The digested and labeled peptide mixture was separated into 10 fractions and subsequently analyzed by nanoLC-RP-MS/MS. As shown in Fig. 1c, a total of 3654 unique proteins were identified with high confidence (false discovery rate < 1 %), in which 3100 proteins were identified with at least two peptide fragments. Among them, 3432 proteins were quantifiable in which 2978 proteins were quantifiable with more than two peptide fragments. 2587 out of 3654 proteins were seen in two technical runs, which indicate the highly reproducible results of this study.

**Fig. 3:**
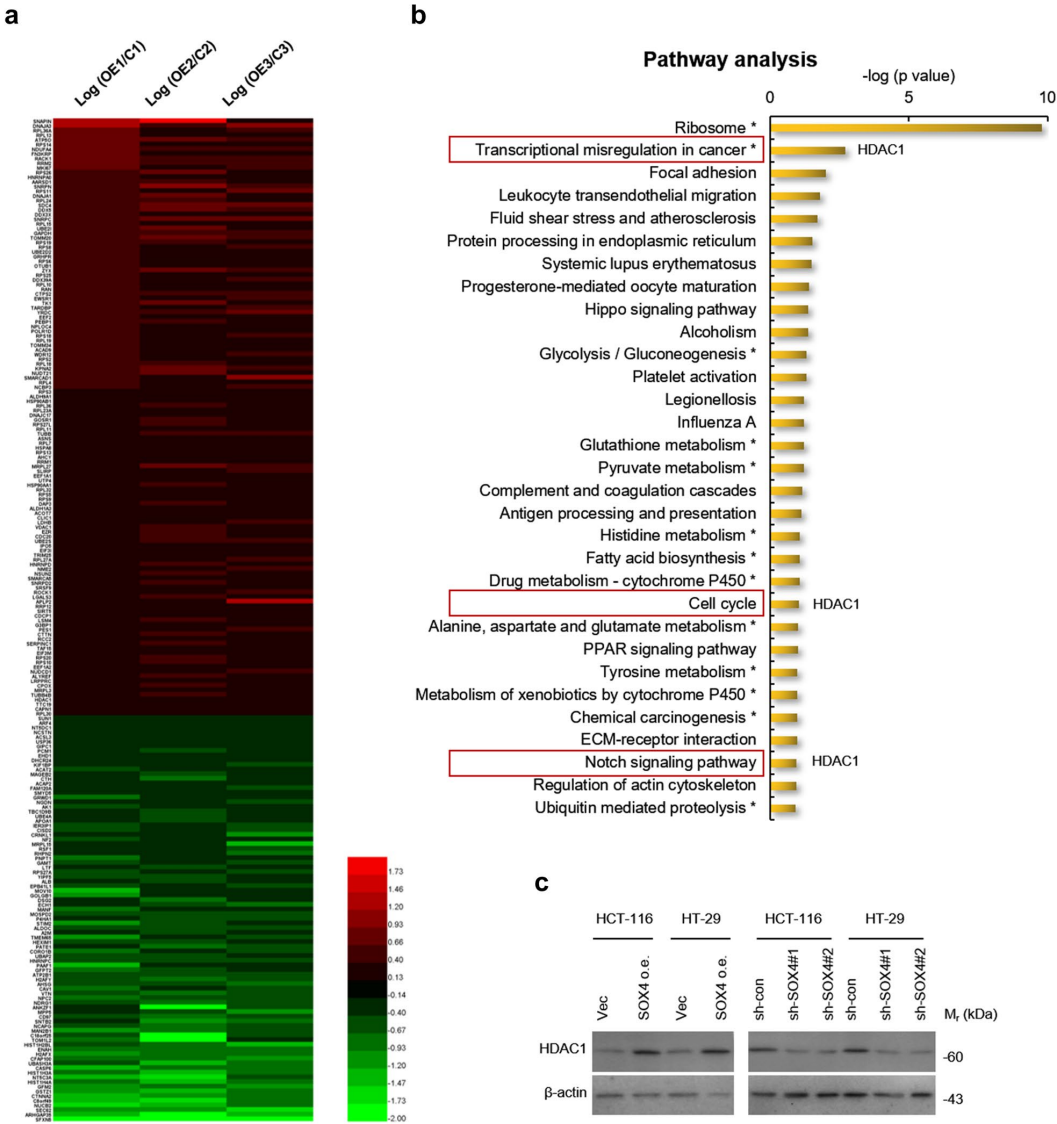
Differentially expressed proteins in SOX4-overexpressing HCT-116 cells and pathway analysis.** a** The data from NanoLC-MS/MS analysis was subjected to quantitative analysis using stringent criteria. Unqualified data with the coefficient of variation more than 30 % between two technical replicates were excluded and the differentially expressed proteins were filtered with cutoff 1.5. In total, 215 differentially expressed proteins (128 upregulated, 87 downregulated) in SOX4-overexpressing HCT-116 cells were identified. OE: SOX4-overexpressing cells, C: Control cells with empty vector transfection. **b** The differentially expressed proteins in SOX4-overexpressing HCT-116 were subjected to iPathwayGuide online software for pathway enrichment and the results were listed. **c** SOX4 positively regulates the protein level of HDAC1 in colorectal cancer cells. The protein levels of HDAC1 in SOX4-overexpressing or SOX4-knockdown HCT-116 and HT-29 cells and their control cells identified by western blot

A stringent criterion was applied for quantitative analysis (Fig. 1b), in which unqualified data with the coefficient of variation more than 30 % between two technical replicates were excluded and the differentially expressed proteins (DEPs) were filtered with cutoff 1.5. Finally, a total of 215 DEPs were identified in SOX4-overexpressing HCT-116 cells, which contained 128 upregulated and 87 downregulated proteins (Fig. 3a and Additional file 1: Table S3).

### Bioinformatic analysis of SOX4-regualted proteins and target verification

To understand the underlying mechanisms reflected by SOX4-induced proteome changes, we performed pathway and Gene Ontology (GO) analysis using iPathwayGuide online software. As shown in Fig. 3b, pathway analysis showed that several important pathways associated with stem cell maintenance were impacted by DEPs, including ‘Hippo’, ‘Notch’, ‘cell cycle’ as well as ‘PPAR’, etc. GO analysis revealed that the DEPs mainly enriched in biological processes (BP), including ‘cotranslational protein targeting to membrane’, ‘translational initiation’, ‘protein targeting to ER’, and ‘viral gene expression’, molecular functions (MF), including ‘nucleic acid binding’, ‘kinase binding’ and ‘histone deacetylase binding’, cellular components (CC), including ‘ribosome’, ‘cytosolic part’, ‘intracellular ribonucleoprotein complex’, and ‘adherents junction’ (Additional file 1: Fig. S2).

**Fig. 4:**
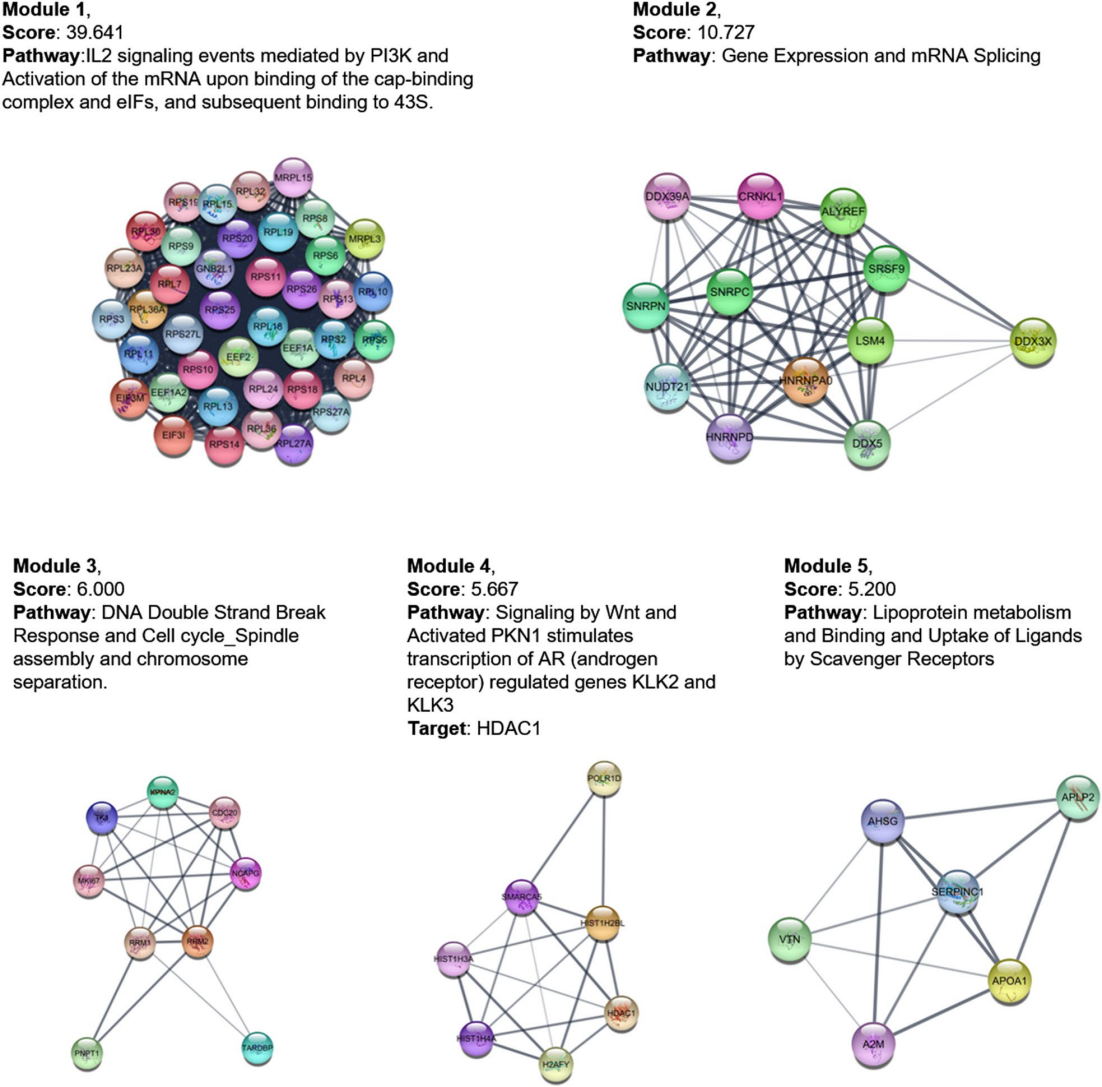
Modules identified from PPI network. The modules were identified using MCODE in Cytoscape, when “MCODE score > 5” was defined as the cutoff criterion

Next, to gain better insight on the DEPs, a protein-protein interaction (PPI) network was constructed based on STRING database, followed by visualization in Cytoscape (Additional file 1: Fig. S3). Subsequently, 5 modules were identified from PPI network using MCODE in Cytoscape, when “MCODE score > 5” was defined as the cutoff criterion (Additional file 1: Table S4 and Fig. 4). Notably, ‘Module 4’ associates with Wnt signaling which is essential for stem cell maintenance.

Through above analysis, we noticed that HDAC1, identified in ‘Module 4’ from PPI network,which involves in Wnt signaling, also plays critical roles in three stemness-associated pathways identified in pathway analysis: ‘Notch’, ‘cell cycle’, and ‘transcriptional misregulation in cancer’ (Fig. 3b). We thus speculated that HDAC1 may play a predominant role in mediating SOX4-induced stemness maintenance of CSCs.

While, to illustrate, we first verified the proteomics results that HDAC1 is positively regulated by SOX4 in CRC cells. As shown in Fig. 3c, by western blot assay, we found that HDAC1 is significantly upregulated in SOX4-overexpressing HCT-116 and HT-29 cells. In contrast, in SOX4-knockdown HCT-116 and HT-29 cells, which were established by lentivirus gene delivery (Additional file 1: Fig. S4), a significant reduction in HDAC1 protein level was observed (Fig. 3c), indicating that HDAC1 is positively regulated by SOX4 in CRC cells.

### HDAC1 is necessary for SOX4 maintaining CRC-SCs stemness

**Fig. 5:**
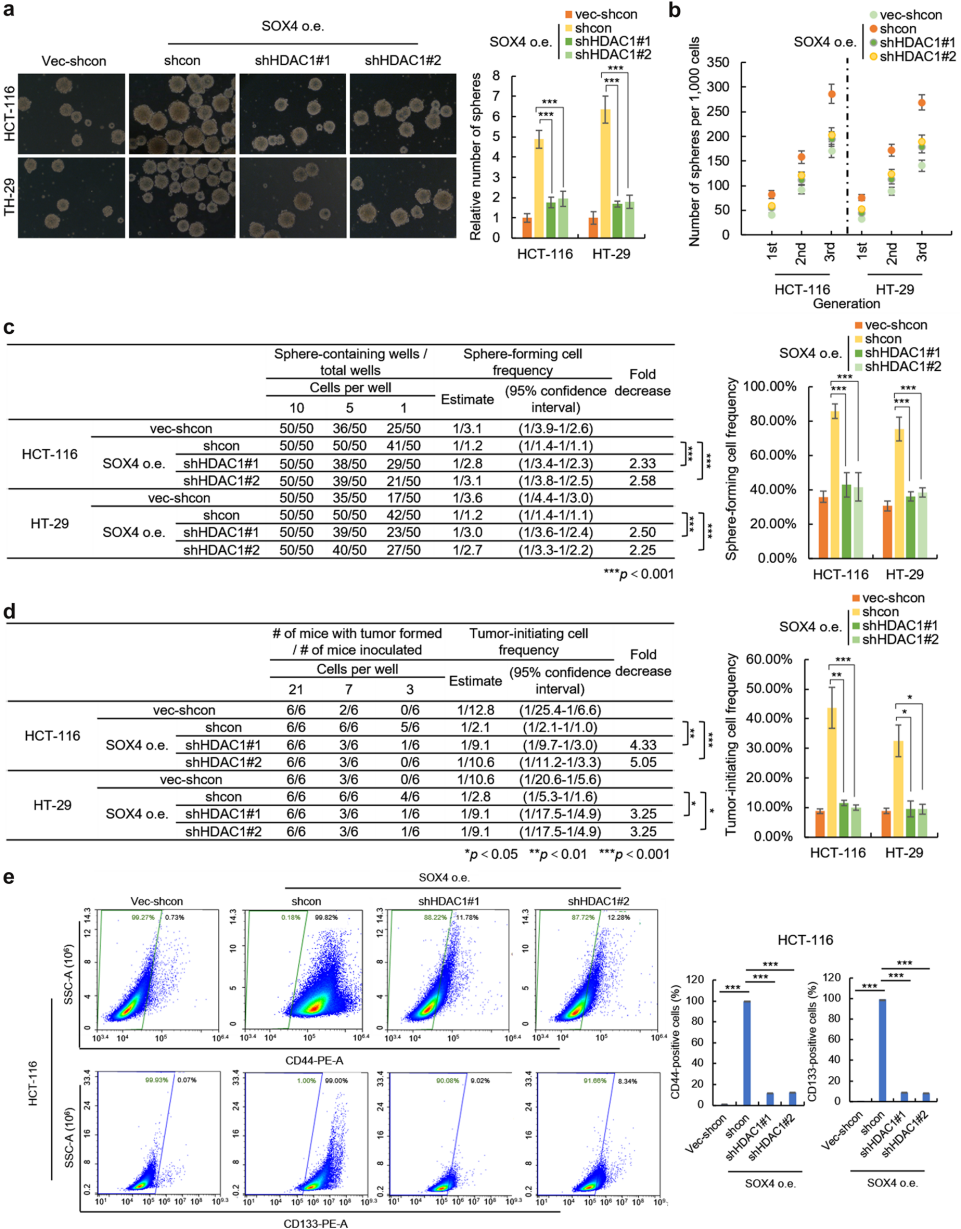
HDAC1 is necessary for SOX4 promoting colorectal cancer stemness. **a** HDAC1 is necessary for SOX4 promoting sphere-forming capacity. Indicated cells were seeded in ultra-low attachment 6 well plate. The number of spheres was counted after 10 days. **b** HDAC1 depletion abolished the effect of SOX4 on the self-renewal capacity of colorectal cancer cells on serial passage. The sphere number of indicated primary, secondary, and tertiary passaged cells was counted after 10 days. **c** The depletion of HDAC1 abolished the effect of SOX4 on the sphere-forming cell frequency of colorectal cancer cells. Indicated cells were maintained in 96-well U-bottomed culture plates at a density of 10, 5 or 1 cells per well and cultured for 10 days. The sphere-forming cell frequency was calculated by ELDA software. **d** Knockdown of HDAC1 attenuated the effect of SOX4 on tumor-initiating cell frequency of colorectal cancer cells. Indicated cells were injected into the subcutaneous tissues of 6-week-old nude mice at a density of 21, 7, 3 cells per mouse. The number of mice developed tumors was counted after 35 days. The tumor-forming cell frequency was calculated by ELDA software. **e** Knockdown of HDAC1 attenuated the effect of SOX4 on the expression of cancer stem cell markers of colorectal cancer cells. The surface protein levels of CD44 and CD133 in indicated HCT-116 cells were analyzed by flow cytometry. Data are represented as mean ± s.d.; *P<0.05, **P<0.01, ***P<0.001; two-tailed Student’s *t*-test

To confirm the critical role of HDAC1 for SOX4 maintaining CRC-SCs stemness, we performed a rescue experiment by depletion of HDAC1 with two independent shRNAs delivered by lentivirus plasmid in SOX4-overexpressing HCT-116 and HT-29 cells (Additional file 1: Fig. S5a). As we expected, the stimulatory effects of SOX4 on all stem-like properties in HCT-116 and HT-29 cells were significantly abolished by HDAC1 depletion, including in vitro sphere-forming capacity (Fig. 5a), self-renewal capacity (Fig. 5b) the frequency of sphere-forming cells in vitro (Fig. 5c and Additional file 1: Fig. S5b), the frequency of tumor-initiating cells in vivo (Fig. 5d and Additional file 1: Fig. S5c) and the expression of CRC-SCs markers (Fig. S5d and Fig. 5e), which confirmed that HDAC1 plays a primary role in mediating SOX4-induced stemness of CRC cells.

### HDAC1 hallmarks CRC-SCs

**Fig. 6:**
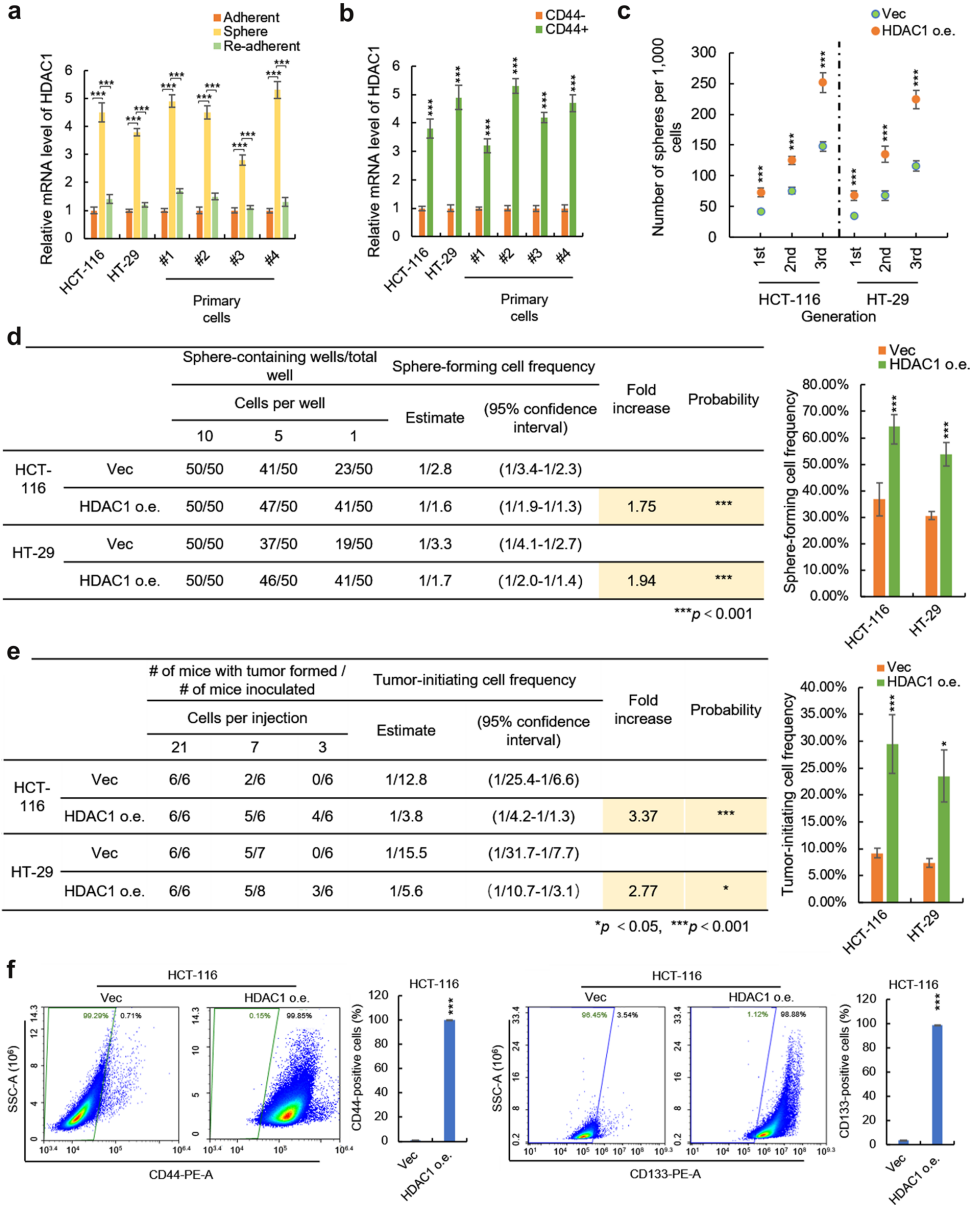
HDAC1 hallmarks colorectal cancer stem cells. **a** HDAC1 is upregulated in colorectal cancer spheres. The HCT-116 and HT-29 and the primary human colorectal cancer cells were adherent cultured in 6-well plate or suspension cultured in an ultra-low 6-well plate. The spheres from suspension cultured plate were then re-adherent cultured in 6-well plate. The total RNA from these cells was isolated and subjected to qRT-PCR analysis. **b** HDAC1 is upregulated in CD44 + colorectal cancer cells. CD44 + and CD44- cells were isolated from HCT-116 and HT-29 and primary human colorectal cancer cells by magnetic-activated cell sorting (MACS). The mRNA levels of HDAC1 in CD44 + or CD44- cells were determined by qRT-PCR. **c** HDAC1 promotes the self-renewal capacity of colorectal cancer cells on serial passage. The HCT-116 and HT-29 cells with or without HDAC1 overexpression were suspension cultured in ultra-low 6 well plates and the sphere number of indicated primary, secondary and tertiary passaged cells was counted after 10 days. **d** HDAC1 promotes the sphere-forming cell frequency of colorectal cancer cells. HCT-116 and HT-29 cells transfected with lentivirus containing HDAC1-overexpressing plasmid or empty control plasmids were seeded into 96-well U-bottomed culture plates at a density of 10, 5 or 1 cells per well and cultured for 10 days. The sphere-forming cell frequency was calculated by ELDA software. **e** HDAC1 promotes tumor-initiating cell frequency of colorectal cancer cells. HCT-116 and HT-29 cells transfected with lentivirus containing HDAC1-overexpressing plasmid or empty control plasmids were injected into the subcutaneous tissues of 6-week-old nude mice at a density of 21, 7, 3 cells per mouse. The number of mice developed tumors was counted after 35 days. The tumor-initiating cell frequency was calculated by ELDA software. **f** HDAC1 promotes the expression of cancer stem cell markers of colorectal cancer cells. The surface protein levels of CD44 and CD133 in HDAC1-overexpressing and control HCT-116 cells were analyzed by flow cytometry. Data are represented as mean ± s.d.; *P<0.05, **P<0.01, ***P<0.001; two-tailed Student’s t-test

Even exhausted studies in non-stem-tumor-cells (nsTCs), the role of HDAC1 in CRC-SCs maintenance has not received enough attention over the past years. To further confirm the critical role of HDAC1 in SOX4 supporting CRC stemness, we next investigated the relationship between HDAC1 and CRC-SCs, and examined whether overexpression of HDAC1 in CRC cells could produce a similar effect with SOX4 overexpression. We first examined the expression level of HDAC1 in suspension-cultured spheres (model of CRC-SCs) and adherent cells (model of nsTCs) as well as re-adherent cells (model of differentiated cancer cells) derived from both HCT-116 and HT-29 cell lines and primary cultured CRC cells. As shown in Fig. 6a, results from qRT-PCR showed that the mRNA level of HDAC1 was significantly upregulated in suspension-cultured spheres compared to adherent and re-adherent cells. Similarly, we isolated CD44 + cells by MACS and found that both CD44 + cells exhibited a higher level of HDAC1 compared to CD44- cells (Fig. 6b), indicating the potential CRC-SCs-specific function for HDAC1.

Next, we established HDAC1-overexpressing HCT-116 and HT-29 cells (Additional file 1: Fig. S6a) and found that HDAC1 overexpression leads to similar results with SOX4 in HCT-116 and HT-29 cells, including enhancement of spere-forming and self-renewal capacities (Fig. 6c), increase in the frequency of sphere-forming (Fig. 6d and Additional file 1: Fig. S6b) and tumor-initiating cells (Fig. 6e and Additional file 1: Fig. S6c) as well as elevation of CRC-SCs markers (Additional file 1: Fig. S6d and Fig. 6f). Furthermore, by Kaplan-meier analysis of TCGA colon adenocarcinoma patients’ gene expression data, we found that high expression of HDAC1 predicts poor prognosis of colon cancer (Additional file 1: Fig. S6e). Taken together, the above results identified that HDAC1 is able to hallmark CRC-SCs and plays a critical role in SOX4 supporting the stemness of CRC cancer.

### SOX4 transcriptionally activates HDAC1 in multiple types of cancer

**Fig. 7:**
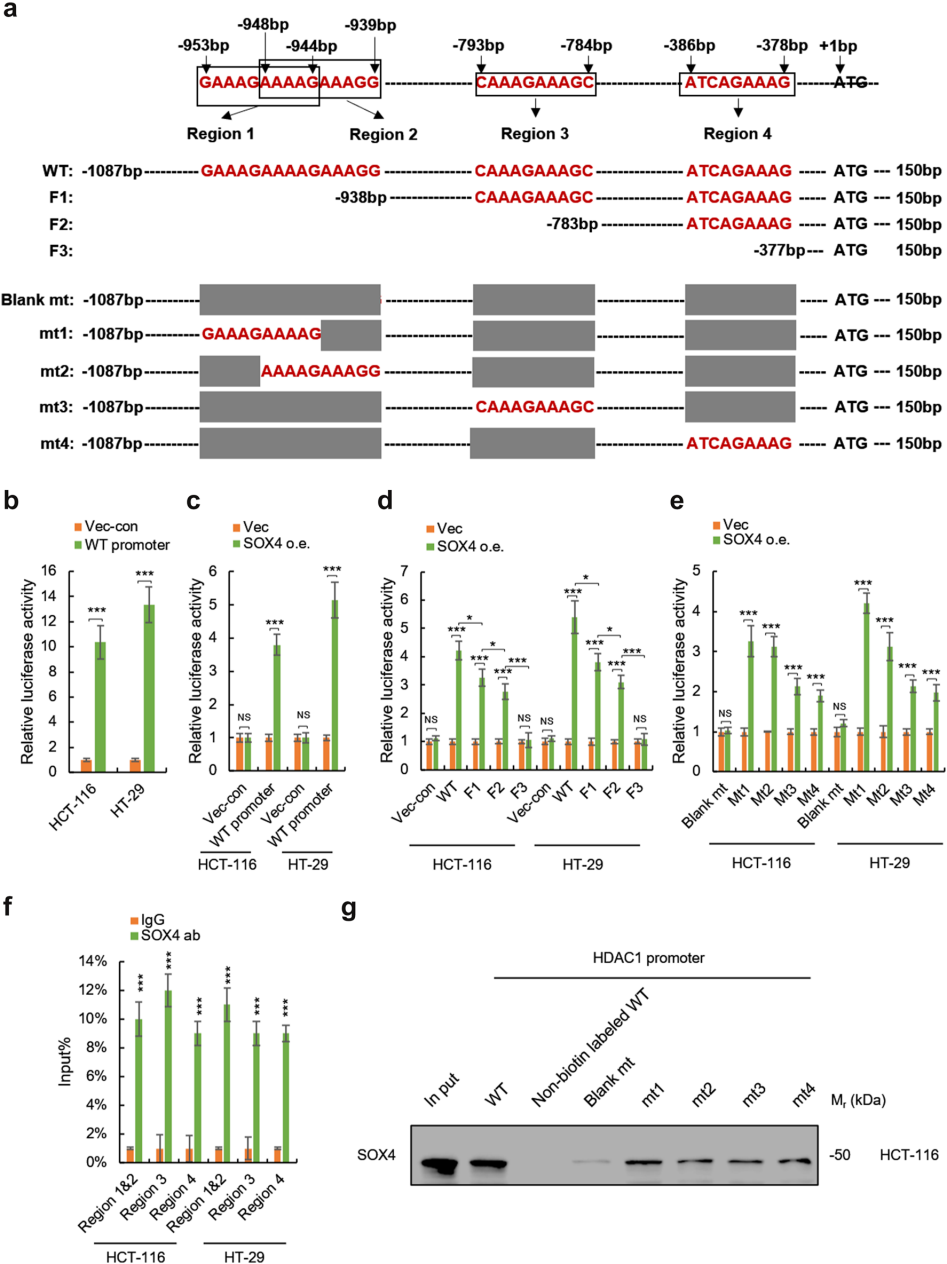
SOX4 transcriptionally activates HDAC1 in multiple types of cancer.** a** Prediction of SOX4 binding sites at the promoter of HDAC1 and the design of four HDAC1 promoter fragments and five mutated HDAC1 promoters for active binding sites analysis. **b** The transcriptional activity of HDAC1 promoter in HCT-116 and HT-29 cells was analyzed by luciferase reporter assay. **c** SOX4 promotes the transcriptional activity of HDAC1 promoter in HCT-116 and HT-29 cells. The luciferase reporter plasmids driven by HDAC1 promoter were transfected into SOX4-overexpressing or control HCT-116 and HT-29 cells. The luciferase activity in these cells was determined by Dual-luciferase Reporter Assay. **d** The transcriptional activities of indicated fragments of HDAC1 promoter in SOX4-overexpressing and control HCT-116 and HT-29 cells were analyzed. **e** The transcriptional activities of indicated mutated HDAC1 promoters in SOX4-overexpressing and control HCT-116 and HT-29 cells were analyzed. **f**,** g** SOX4 directly binds to the promoter of HDAC1 in colorectal cancer cells. **f** ChIP-PCR was employed to determine the interaction between SOX4 protein and HDAC1 promoter. **g** DNA pull-down assay was performed to determine the interaction between indicated DNA fragments and SOX4 protein. Data are represented as mean ± s.d.; *P<0.05, **P<0.01, ***P<0.001; two-tailed Student’s *t*-test

We next investigated the mechanism underlying SOX4 regulating HDAC1. As SOX4 primarily functions as a transcription factor, we speculated that SOX4 could directly regulate HDAC1 at the transcriptional level. To investigate this, we first examined the mRNA level of HDAC1 in SOX4-overexpressing HCT-116 and HT-29 cells. As shown in Additional file 1: Fig. S7a, ectopic expression of SOX4 significantly increased the mRNA level of HDAC1 in both HCT-116 and HT-29 cells, indicating that SOX4 may transcriptionally activate HDAC1. Next, we predicted the potential binding sites of SOX4 in the promoter of HDAC1 according to stephin J Vervoort and Christopher D. Scharer and their colleagues’ publications [37, 39] (Additional file 1: Fig. S7b) and found that there are four potential SOX4 binding sites, named region 1 (-953 bp to -944 bp), region 2 (-948 bp to -939 bp), region 3 (-793 bp -784 bp) and region 4 (-386 bp to -378 bp), and region 1 and region 2 are overlapped (Fig. 7A). We next prepared four HDAC1 promoter fragments: WT (wild type), F1 (without region 1 and 2), F2 (without region 3) and F3 (without region 4), and five mutated HDAC1 promoters: blank mt (all regions were removed), mt1 (with only region 1), mt2 (with only region 2), mt3 (with only region 3) and mt4 (with only region 4) (Fig. 7a). Then, luciferase reporter assay was employed to examine the SOX4 response of these HDAC1 promoters. As shown in Fig. 7b, the HCT-116 and HT-29 cells transfected with WT HDAC1 promoter-driven luciferase reporter plasmids showed elevated luciferase activity, indicating that the reaction system is well constructed. Next, we found that SOX4-overexpresisng HCT-116 and HT-29 cells transfected with WT HDAC1 promoter-driven plasmids showed upregulated luciferase activity, compared to cells transfected empty control vectors (Fig. 7c), indicating that SOX4 activates the transcriptional activity of HDAC1 promoter. Surprisingly, we found that all F1, F2 and F3 fragments of HDAC1 promoter showed significantly reduced sensitivity to SOX4 in both HCT-116 and HT-29 cells (Fig. 7d), which indicated that all regions 1–4 are responsible for the effect of SOX4 on HDAC1 promoter. Furthermore, there is no stimulatory effect of SOX4 on blank mutated HDAC1 promoter was detected and addition of regions 1–4 can lead to a significant response of SOX4 for blank mutated HDAC1 promoter (Fig. 7e), which confirmed that all regions 1–4 are responsible for the response of HDAC1 promoter to SOX4.

To demonstrate the direct binding between SOX4 and HDAC1 promoter, we performed ChIP-PCR assay. As shown in Fig. 7f, all region 1, 2, 3 and 4 were detected by qRT-PCR from the DNA fragments eluted from SOX4-bound beads, which indicated the direct binding between SOX4 and HDAC1 promoter. Furthermore, we performed DNA pull-down assay with WT and mutated HDAC1 promoter. We found that Wt and m1–4 mutated, but not blank, HDAC1 promoters showed a significant binding with SOX4 (Fig. 7g), which confirmed that SOX4 directly binds to HDAC1 promoter and regions 1–4 are responsible for this process.

Next, we wonder whether SOX4-HDAC1 axis is conserved in multiple types of cancer. The SOX4-overexpressing ovarian, lung, gastric, liver, and breast cancer cells were established by lentivirus gene delivery system (Additional file 1: Fig. S7c). We found that SOX4 was able to upregulate the mRNA level of HDAC1 (Additional file 1: Fig. S7d) and the transcriptional activity of HDAC1 promoter (Additional file 1: Fig. S7e) in all tested cells. Similarly, SOX4 binds to the promoter of HDAC1 in all types of tested cells (Fig. S7F), indicating the mechanism that SOX4 transcriptionally activates HDAC1 is conserved in multiple types of cancer.

Collectively, the above results demonstrated that SOX4 supports cancer stemness by transcriptionally activates HDAC1.

## Discussion

In this study, we confirmed the stimulatory effect of SOX4 on the stemness of CRC cells, identified SOX4-induced proteome changes in CRC cells, and proved that HDAC1 serves as a prime downstream target of SOX4 that mediates SOX4-induced CRC stemness. Furthermore, we showed that SOX4-HDAC1 axis is conserved in multiple types of cancer cells. This study thus explores a novel mechanism that SOX4 promotes cancer stemness and suggests that HDAC1 inhibition would be an effective strategy for eradicating human CSCs driven by SOX4 overexpression.

SOX4 is an important drug target for cancer therapy. First, several studies have reported that SOX4 inhibition by genetic manipulation suppresses the proliferation and metastasis of cancer cells in vitro and in vivo [40, 41]. Moreover, the tight relationship between SOX4 and EMT has been explored in the past decade [42]. Furthermore, in this study, we confirmed the stimulatory effect of SOX4 on CRC cancer stemness (Fig. 2), which demonstrated that targeting SOX4 holds the promise to eradicate not only normal differentiated cancer cells, but also undifferentiated CRC cells.

Development of the strategies suppressing transcription factors, such as SOX4, is more difficult compared to traditional drug targets with enzymatic activity. Transcription factors do not have enzymatic activity, and thus lacking binding pockets for molecule design [43]. Take SOX family proteins as an example, as far as we know, despite massive studies, only one small molecular inhibitor, Sm4, a natural product from brown algae *caulocystis cephalornithos*, targeting SOX18, has been developed by high throughput drug screening, which blocks HMG domain-mediated SOX18-RBPJ interaction [44]. While its safety and feasibility for clinical use still need to be studied. Alternatively, suppressing the prime downstream target of SOX4 is a feasible strategy to block signaling delivered from SOX4. To achieve this, several studies have performed to investigate the downstream target of SOX4 in various types of cells, such as small cell lung cancer cells, acute lymphoblastic leukemia cells, etc. [23–26]. However, these studies were conducted in the cells that were cultured under normal conditions and because that CSCs are a specific subtype of cancer cells with distinct phenotype, function and metabolic features, the function of a certain gene should be differentially regulated in the specific cellular context of CSCs [1–3]. While, to our knowledge, the omics studies of SOX4 in CSCs have not been performed yet. In addition, the SOX4-drived proteome changes have not been revealed, which would be more important for communication between CSCs and the environment for stemness maintenance.

By proteomics study, we found that, in CRC spheres, SOX4 regulates several important signaling pathways involved in stemness maintenance for both normal and cancer stem cells, including Hippo signaling pathway, cell cycle, PPAR signaling pathway, and Notch signaling pathway, etc. (Fig. 3b). Hippo signaling controls organ size and plays important roles in stem cell fate decision [45]. The upregulated activity of YAP, the prime output of Hippo signaling, has been identified in various types of cancer cells including CSCs [45]. Our results showed that Hippo signaling pathway is one of the downstream targets of SOX4 in CRC-SCs, and thus should be a potential drug target for suppressing SOX4 in CSCs.

Controlling the symmetric/asymmetric division is a specific ability conserved for stem cells to survive and expand [46]. In CSCs, the mechanisms underlying the regulation of cell cycle in response to their microenvironment remained elusive. Usually, the transcription factors play an important role during this kind of communication [47] and our proteomics results showed that the cell cycle is a potential downstream target of SOX4, which indicated that SOX4 may be associated with the regulation of the division of CSCs to support the stemness.

The peroxisome proliferator-activated receptor (PPAR) nuclear receptors are composed of three family members: PPARα, PPARβ, and PPARγ and evidence has shown their context-specific oncogenic and tumor-suppressive roles [48]. For example, PPARγ maintains the stemness in ERBB2-positvie breast CSCs [49], in contrast, PPARγ agonists inhibit the expansion of brain tumor stem cells [50]. Our proteomics results showed that PPAR signaling pathway is a downstream effector of SOX4, which indicates that SOX4 may be a master regulator for the context-specific role of PPAR family members. Although the mechanisms underlying crosstalk between SOX4 and PPAR signaling in response to CSCs microenvironments still need further studies, our study provided novel insight into the downstream targets of SOX4 in CSCs and the potential targets for SOX4 suppression.

Epigenetic modifications play essential roles in cell fate decision through the regulation of transcription at the genome level [38]. HDAC1 is a key epigenetic regulator that governs the transcription of oncogenes and tumor-suppressors by reversing acetylation of histone proteins [14]. Accumulation evidence has shown the oncogenic role of HDAC1 and several small molecular inhibitors targeting HDAC1 have been developed [20]. While the role of HDAC1 in CRCs and its regulation are not completely understood. Our bioinformatics study showed that HDAC1 may mediate the regulatory effect of SOX4 on several important signaling pathways associated with stem cell maintenance, including Wnt (Fig. 4, module 4), PPAR, Notch and transcriptional misregulation in cancer (Fig. 3b). Subsequent experiments confirmed that HDAC1 hallmarks CRC-SCs (Fig. 6) and is at least partially necessary for SOX4 promoting the stemness of CRC cells (Fig. 5). Furthermore, we also found that SOX4 directly binds to HDAC1 promoter and this SOX4-HDAC1 axis is conserved in multiple types of cancer cells (Fig. 7). These results thus reveal a novel mechanism that HDAC1 is a prime mediator for SOX4 supporting cancer stemness and suggest the potential therapeutic application of HDAC1 inhibition for eradicating SOX4-driven CSCs.

## Conclusions

In summary, our results demonstrate that transcriptional activation of HDAC1 is the primary mechanism underlying SOX4 supporting cancer stemness and this finding suggests that inhibition of HDAC1 would be an effective therapeutic strategy for human cancer with aberrant SOX4 upregulation.

## Supplementary Information

**Additional file 1: Figures S1-S5.** Showing characterization of SOX4-overexpressing CRC cells, the stimulatory effect of SOX4 on the frequency of sphere-forming and tumor-initiating CRC cells, GO analysis of differentially expressed proteins, PPI network of differentially expressed proteins, characterization of SOX4-knockdown CRC cells, characterization of SOX4-overexpressing HDAC1-knockdown CRC cells, the necessary role of HDAC1 for SOX4 promoting CSCs markers, characterization of HDAC1-overexpressing CRC cells, the stimulatory effect of HDAC1 on the expression of CSCs markers, HDAC1 predicts poor prognosis of colorectal cancer patients, SOX4 transcriptionally activates HDAC1 by directly binding to HDAC1 promoter. **Tables S1-S4.** Showing the primers and antibodies used in this study as well as differentially expressed proteins identified by proteomics study, top 5 PPI network modules

## Data Availability

All mass spectrometry proteomics data have been deposited to the ProteomeXchange Consortium via the PRIDE partner repository with the data set identifier PXD019694. Other data are available from the corresponding author.
