# Supplementary material for: SOX4 maintains the stemness of cancer cells via transcriptionally enhancing HDAC1 revealed by comparative proteomics study
Source: Cell Biosci. 2021 Jan 22;11:23. doi: 10.1186/s13578-021-00539-y (PMC7821488; doi:10.1186/s13578-021-00539-y)
Supplement: Supplementary file 1 — Additional file 1: Figures S1-S5. Showing characterization of SOX4-overexpressing CRC cells, the stimulatory effect of SOX4 on the frequency of sphere-forming and tumor-initiating CRC cells, GO analysis of differentially expressed proteins, PPI network of differentially expressed proteins, characterization of SOX4-knockdown CRC cells, characterization of SOX4-overexpressing HDAC1-knockdown CRC cells, the necessary role of HDAC1 for SOX4 promoting CSCs markers, characterization of HDAC1-overexpressing CRC cells, the stimulatory effect of HDAC1 on the expression of CSCs markers, HDAC1 predicts poor prognosis of colorectal cancer patients, SOX4 transcriptionally activates HDAC1 by directly binding to HDAC1 promoter. Tables S1-S4. Showing the primers and antibodies used in this study as well as differentially expressed proteins identified by proteomics study, top 5 PPI network modules [file 13578_2021_539_MOESM1_ESM.docx]

Supporting information

**SOX4 maintains the stemness of cancer cells via transcriptionally enhancing HDAC1 revealed by comparative proteomics study**

Jingshu Liu^1,2,3,4^, Jiangfeng Qiu^5^, Zhiqi Zhang^6^, Lei Zhou^7,8,9^, Yunzhe Li^1,2^, Dongyan Ding^1,2^, Yang Zhang^10^, Dongling Zou^2^, Dong Wang^2^, Qi Zhou^1,2,3,4*^, Tingyuan Lang^1,2,3,4*^

1 College of Bioengineering, Chongqing University, Chongqing, 400044, People's Republic of China.

2 Department of Gynecologic Oncology, Chongqing University Cancer Hospital, Chongqing, 400030, People's Republic of China.

3 Chongqing Key Laboratory of Translational Research for Cancer Metastasis and Individualized Treatment, Chongqing University Cancer Hospital, Chongqing, 400030, People's Republic of China.

4 Key Laboratory for Biorheological Science and Technology of Ministry of Education (Chongqing University), Chongqing University Cancer Hospital, Chongqing, 400044, People's Republic of China.

5 Department of Gastrointestinal Surgery, Renji Hospital Shanghai Jiao Tong University School of Medicine, Shanghai 200127, People's Republic of China.

6 Department of General Surgery, Shanghai Fourth People’s Hospital Affiliated to Tongji University School of Medicine, Shanghai 200081, People's Republic of China.

7 Singapore Eye Research Institute, The academia, 20 College Road, Discovery Tower Level 6, Singapore, 169856, Singapore.

8 Department of Ophthalmology, Yong Loo Lin School of Medicine, National University of Singapore, Singapore.

9 Ophthalmology and Visual Sciences Academic Clinical Research Program, Duke-NUS Medical School, National University of Singapore, Singapore.

10 Laboratory Department, Chongqing University Cancer Hospital, Chongqing, 400030, People's Republic of China.

***Correspondence:** Qi Zhou ([cqzl_zq@163.com](mailto:cqzl_zq@163.com)) and Tingyuan Lang ([michaellang2009@163.com](mailto:michaellang2009@163.com))

^2^Department of Gynecologic Oncology, Chongqing University Cancer Hospital, Chongqing, 400030, People's Republic of China.

**Supplementary Figures**

**
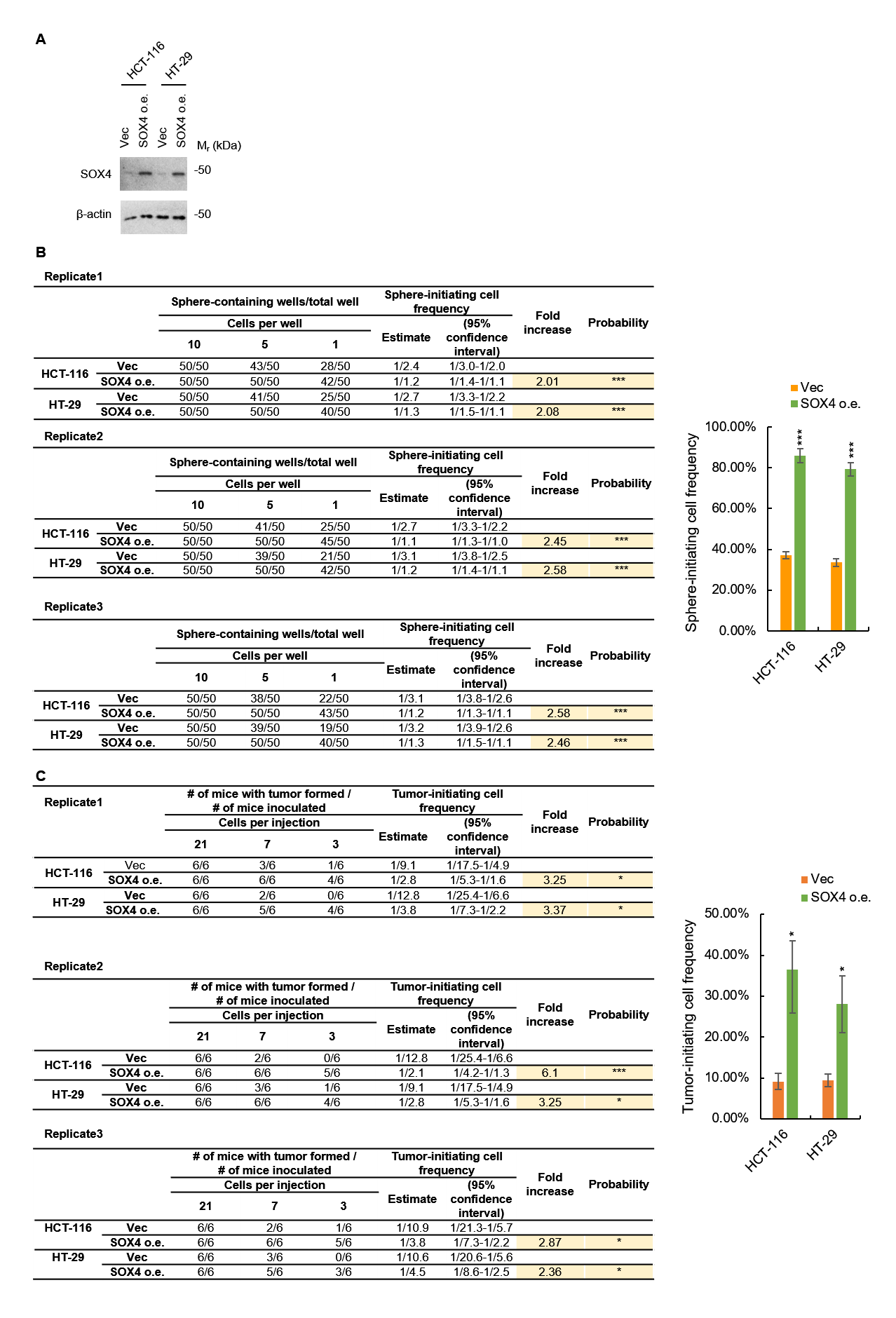
**

**Figure S1. Characterization of SOX4-overexpressing HCT-116 and HT-29 cells and the stimulatory effect of SOX4 on the frequency of sphere-forming and tumor-initiating HCT-116 and HT-29 cells. (A)** The protein levels of SOX4 and internal control (GAPDH) in SOX4-overexpressing HCT-116 and HT-29 cells and their corresponding control cells were characterized by western blot. **(B)** SOX4 promotes sphere-forming cell frequency of colorectal cancer cells. SOX4-overexpressing and control cells were seeded into 96-well U-bottomed culture plates at a density of 10, 5 or 1 cells per well and cultured for 10 days. The sphere-forming cell frequency was calculated by ELDA software. **(C)** SOX4 promotes tumor-initiating cell frequency of colorectal cancer cells. SOX4-overexpressing and control cells were injected into the subcutaneous tissues of 6-week-old nude mice at a density of 21, 7, 3 cells per mouse. The number of mice developed tumors was counted after 35 days. The tumor-forming cell frequency was calculated by ELDA software. Data are represented as mean ± s.d.; **P*＜0.05, ***P*＜0.01, ****P*＜0.001; two-tailed Student’s t-test.

**
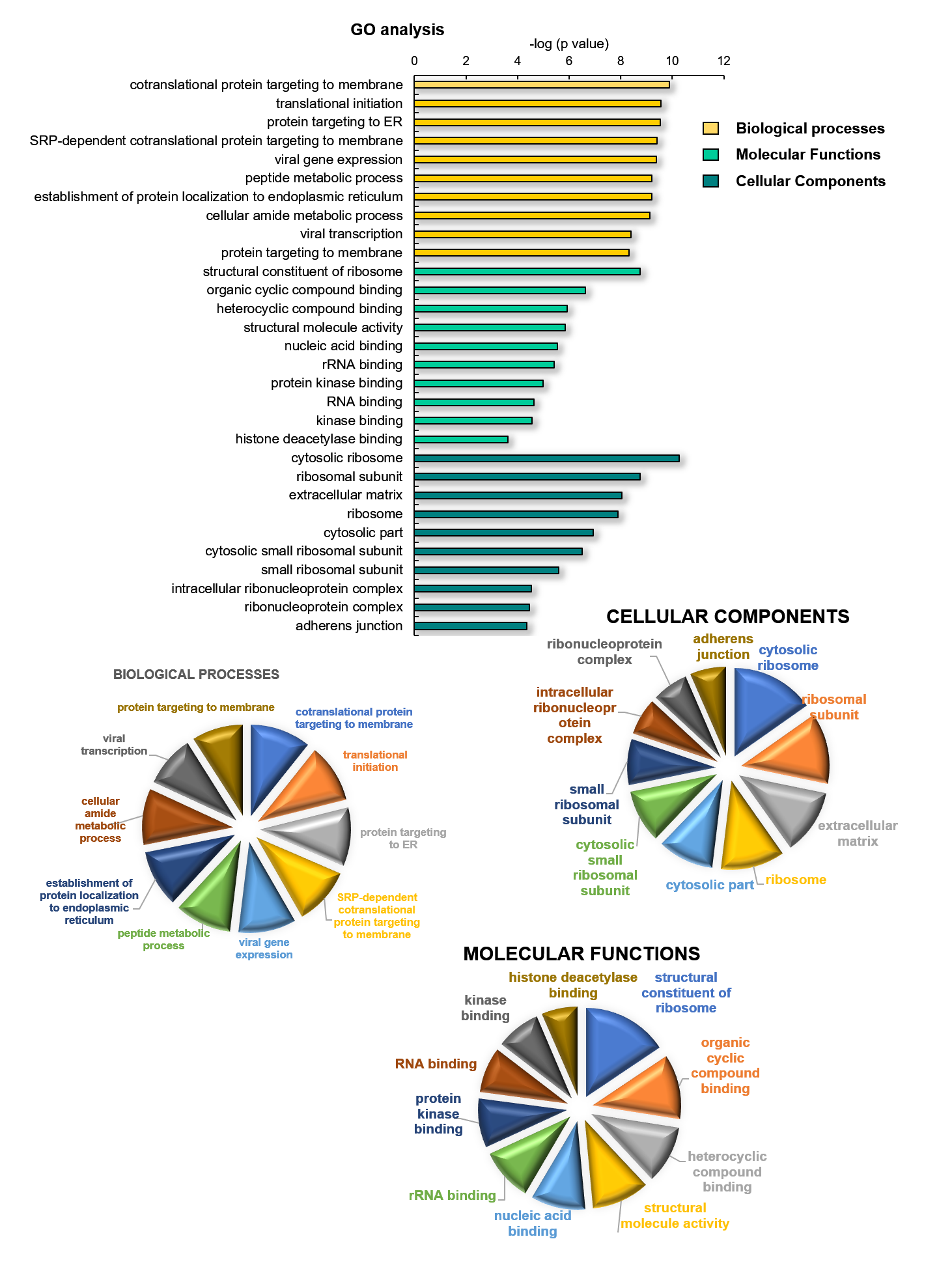
**

**Figure S2. GO analysis of differentially expressed proteins.** The differentially expressed proteins in SOX4-overexpressing HCT-116 were subjected to iPathwayGuide online software for Gene ontology (GO) analysis and the results were presented as bar chart and pie chart.

**
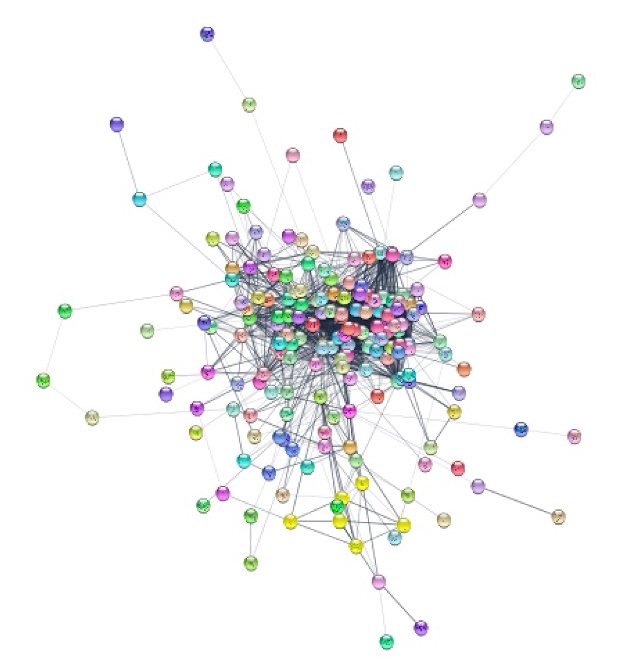
**

**Figure S3. PPI network of differentially expressed proteins.** PPI analysis showed the signalling network between differentially expressed proteins. Node size was continuous mapping based on the degree.

**
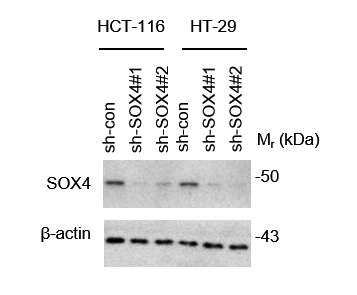
**

**Figure S4. Characterization of SOX4-knockdown HCT-116 and HT-29 cells.** The protein levels of SOX4 and internal control (GAPDH) in SOX4-knockdown HCT-116 and HT-29 cells and their corresponding control cells were characterized by western blot.

**
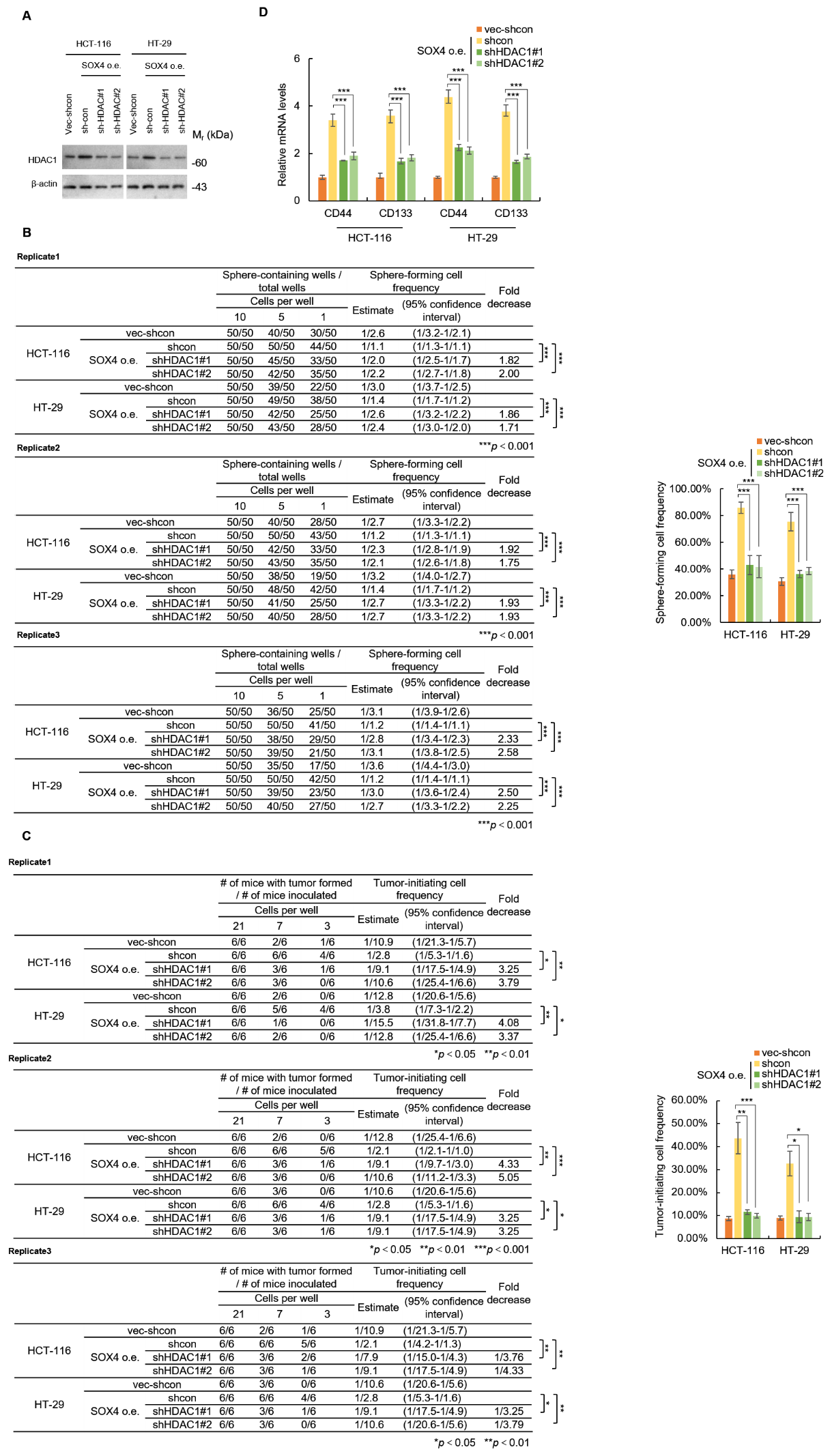
**

**Figure S5. HDAC1 is necessary for SOX4 promoting colorectal cancer stemness (related to Figure 5). (A)** Characterization of SOX4-overexpressing HDAC1-knockdown HCT-116 and HT-29 cells. The protein levels of indicated genes in indicated cells were tested by western blot. **(B)** Depletion of HDAC1 abolished the effect of SOX4 on sphere-forming cell frequency of colorectal cancer cells. Indicated cells were maintained in 96-well U-bottomed culture plates at a density of 10, 5 or 1 cells per well and cultured for 10 days. The sphere-forming cell frequency was calculated by ELDA software. **(D)** Knockdown of HDAC1 attenuated the effect of SOX4 on tumor-initiating cell frequency of colorectal cancer cells. Indicated cells were injected into the subcutaneous tissues of 6-week-old nude mice at a density of 21, 7, 3 cells per mouse. The number of mice developed tumors was counted after 35 days. The tumor-initiating cell frequency was calculated by ELDA software. **(D)** HDAC1 knockdown abolished the stimulatory effect of SOX4 on the expression of CD44 and CD133 in HCT-116 and HT-29 cells. qRT-PCR analysis of CD44 and CD133 in indicated cells. Data are represented as mean ± s.d.; **P*＜0.05, ***P*＜0.01, ****P*＜0.001; two-tailed Student’s t-test.

**
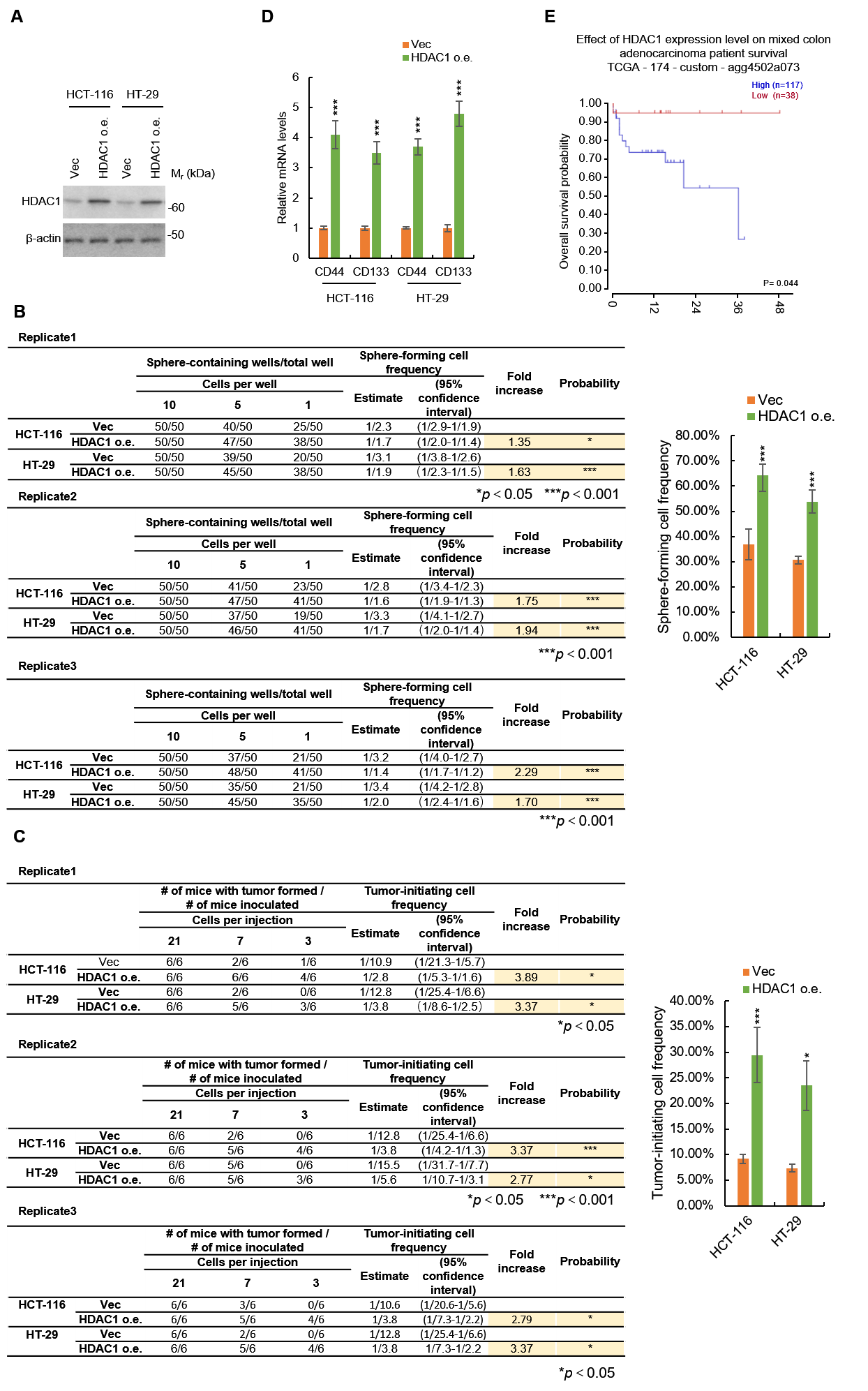
**

**Figure S6. HDAC1 hallmarks colorectal cancer stem cells. (A)** Characterization of HDAC1-overexpressing HCT-116 and HT-29 cells. The protein levels of SOX4 and internal control (GAPDH) in HDAC1-overexpressing HCT-116 and HT-29 cells and their corresponding control cells were characterized by western blot. **(B)** HDAC1 promotes sphere-forming cell frequency of colorectal cancer cells. HCT-116 and HT-29 cells transfected with lentivirus containing HDAC1-overexpressing plasmid or empty control plasmids were seeded into 96-well U-bottomed culture plates at a density of 10, 5 or 1 cells per well and cultured for 10 days. The sphere-forming cell frequency was calculated by ELDA software. **(C)** HDAC1 promotes tumor-initiating cell frequency of colorectal cancer cells. HCT-116 and HT-29 cells transfected with lentivirus containing HDAC1-overexpressing plasmid or empty control plasmids were injected into the subcutaneous tissues of 6-week-old nude mice at a density of 21, 7, 3 cells per mouse. The number of mice developed tumors was counted after 35 days. The tumor-initiating cell frequency was calculated by ELDA software. **(D)** HDAC1 promotes the expression of colorectal cancer stem cell markers in colorectal cancer cells. The mRNA levels of CD44 and CD133 in HCT-116 and HT-29 cells transfected with lentivirus containing HDAC1-overexpressing plasmid or empty control plasmids were analyzed by qRT-PCR. **(E)** High expression of HDAC1 predicts poor prognosis of colorectal cancer patients. The TCGA dataset was downloaded from R2 database. Kaplan-meier analysis was employed to determine the relationship between HDAC1 expression and survival of colorectal cancer patients. Data are represented as mean ± s.d.; **P*＜0.05, ***P*＜0.01, ****P*＜0.001; two-tailed Student’s t-test.

**
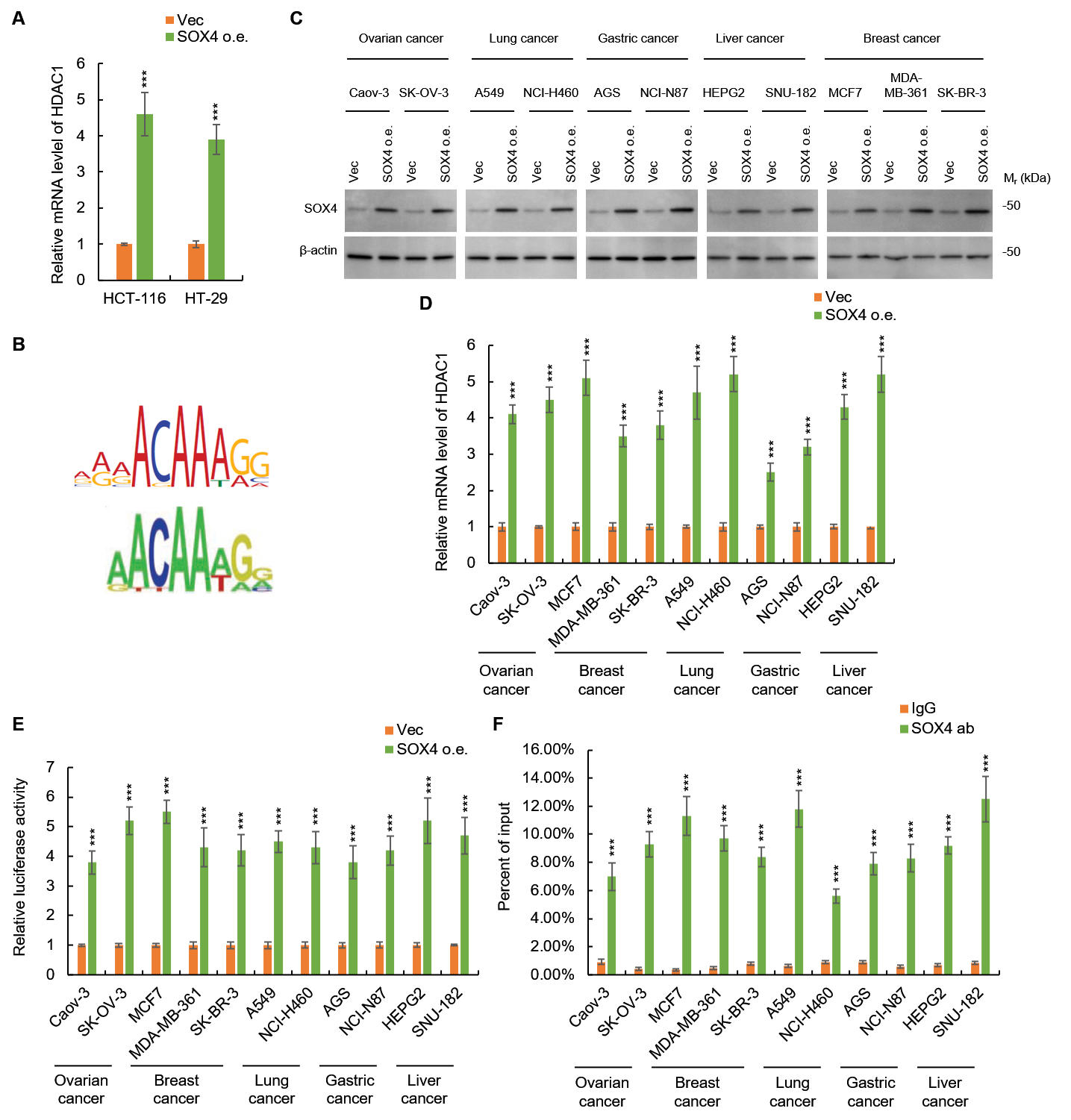
 Figure S7. SOX4 transcriptionally activates HDAC1 (related to Figure 7). (A)** qRT-PCR analysis of HDAC1 mRNA levels in HCT-116 and HT-29 cells transfected with SOX4-overexpressing and control empty vectors. **(B)** The sequence of predicted SOX4-binding site. **(C)** Characterization of SOX4-overexpressing cells. The protein levels of SOX4 and internal control (GAPDH) in indicated SOX4-overexpressing cells and their corresponding control cells were characterized by western blot. **(D-F)** SOX4-HDAC1 axis is conserved in multiple types of cancer. The effect of SOX4 on HDAC1 mRNA level **(D)** and transcriptional activity of HDAC1 promoter **(E)** and the binding between SOX4 and HDAC1 promoter **(F)** in indicated cells were examined by qRT-PCR **(D)**, luciferase reporter assay **(E)** and ChIP assay **(F)**. Data are represented as mean ± s.d.; **P*＜0.05, ***P*＜0.01, ****P*＜0.001; two-tailed Student’s t-test.

**
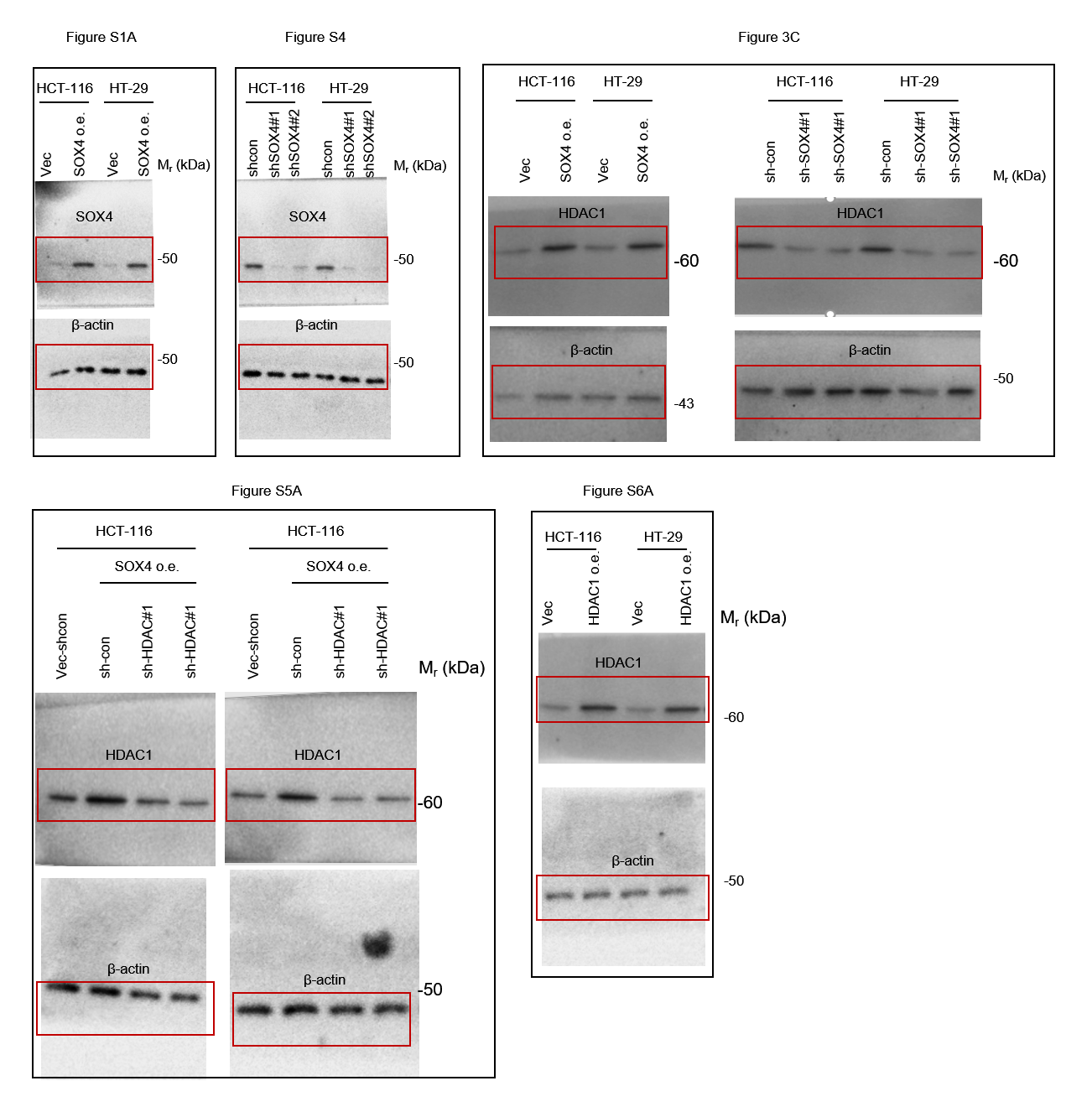
**

**Figure S8. Uncropped gel for Figure S1A, S4, 3C, S5A, S6A.**

**
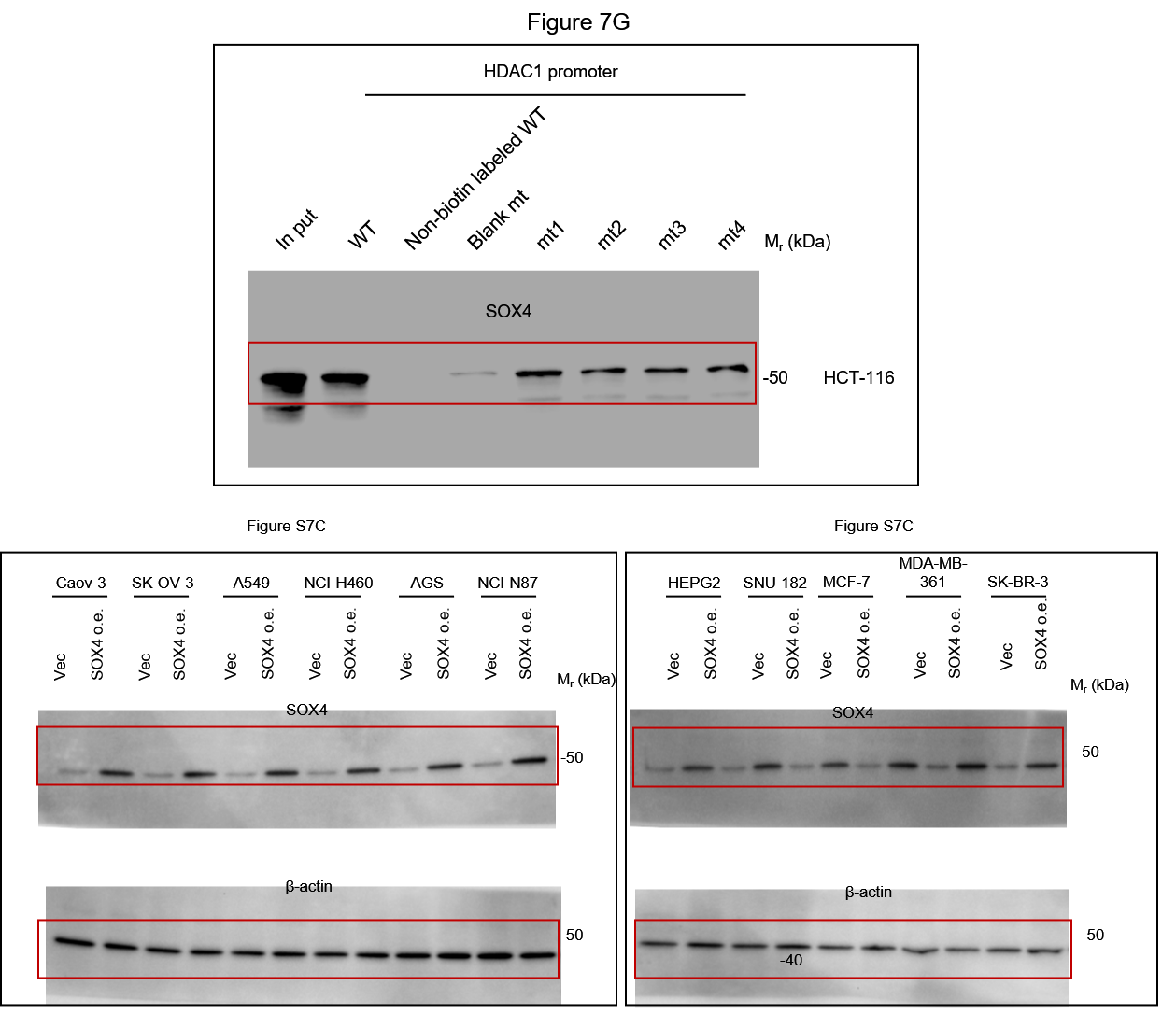
**

**Figure S9. Uncropped gel for Figure 7G and S7.**

**Supplementary Tables**

**Table S1. Primers used in the study.**

| **Reverse transcription PCR** | |
| --- | --- |
| **SOX4 CDs amplification** | F: 5’-ATGGTGCAGCAAACCAACAATGCC-3’ |
|  | R: 5’-TCAGTAGGTGAAAACCAGGTT-3’ |
| **HDAC1 CDs amplification** | F: 5’-ATGGCGCAGACGCAGGGCACCCG-3’ |
|  | R: 5’-TCAGGCCAACTTTGACCTCCTCCTT-3’ |
| **HDAC1 promoter (WT) (-1087 - +150 bp)** | F: 5’-GGAGGCTGAGGCAGGAGAACCG-3’ |
|  | R: 5’-CCTCAGCCTCCCGCAGCCTCCCA-3’ |
| **HDAC1 promoter (F1) (-938 - +150 bp)** | F: 5’-AAACCTGCCCTCCTATCATAGGAT-3’ |
|  | R: 5’-CCTCAGCCTCCCGCAGCCTCCCA-3’ |
| **HDAC1 promoter (F2) (-783 - +150 bp)** | F: 5’-ATTTTAAAAACCCTGTACAATTAA-3’ |
|  | R: 5’-CCTCAGCCTCCCGCAGCCTCCCA-3’ |
| **HDAC1 promoter (F3) (-377 - +150 bp)** | F: 5’-TTGGCCTCCGATCTCCAAGCACGC-3’ |
|  | R: 5’-CCTCAGCCTCCCGCAGCCTCCCA-3’ |
| **Real-Time Quantitative Reverse Transcription PCR** | |
| **CD44** | F: 5’-CTGCCGCTTTGCAGGTGTA-3’ |
|  | R: 5’-CATTGTGGGCAAGGTGCTATT-3’ |
| **CD133** | F: 5’-AGTCGGAAACTGGCAGATAGC-3’ |
|  | R: 5’-GGTAGTGTTGTACTGGGCCAAT-3’ |
| **HDAC1** | F: 5’-CTACTACGACGGGGATGTTGG-3’ |
|  | R: 5’-GAGTCATGCGGATTCGGTGAG-3’ |
| **GAPDH** | F: 5’-GGAGCGAGATCCCTCCAAAAT-3’ |
|  | R: 5’-GGCTGTTGTCATACTTCTCATGG-3’ |
| **Chromatin immunoprecipitation assay** | |
| **HDAC1 promoter region 1&2 (-1087 - -939bp)** | F: 5’-GGAGGCTGAGGCAGGAGAACCG-3’ |
|  | R: 5’-CCTTTCTTTTCTTTCCCTGTT-3’ |
| **HDAC1 promoter region 3 (-793 – 614 bp)** | F: 5’-CAAAGAAAGCATTTTAAAAACCCT-3’ |
|  | R: 5’-AGCGACACTTTACCAATGTTATTT-3’ |
| **HDAC1 promoter region 4 (-386 - +150 bp)** | F: 5’-ATCAGAAAGTTGGCCTCCGATCT-3’ |
|  | R: 5’-CCTCAGCCTCCCGCAGCCTCCCA-3’ |

**Table S2. Antibodies used in this study.**

| **Antigen** | **Antibody** | **Conjugated with** | **Application** | **Manufacture** | **Catalog #** | **Dilution (1:)** |
| --- | --- | --- | --- | --- | --- | --- |
| SOX4 | Rabbit, NA | Unconjugated | WB | Sigma | AV38234 | 1000 |
| SOX4 | Rabbit, NA | Unconjuated | ChIP-PCR | Diagenode | C15310129 | NA |
| HDAC1 | Rabbit IgG | Unconjugated | WB | CST | 34589 | 1000 |
| β-actin | Rabbit, IgG | Unconjugated | WB | CST | 4970 | 1000 |
| CD133 | Mouse, IgG1κ | PE | MACS | Miltenyi Biotec | 130113670 | NA |
| Rabbit IgG | Goat, NA | HRP | WB | CST | 7074 | 1000 |
| CD133 | Mouse, IgG | PE | Flowcytometry | ThermoFisher | 17-1338-42 | 100 |
| CD44 | Rat, IgG | PE | Flowcytometry | ThermoFisher | 12-0441-81 | 100 |
| Isotype control | Rabbit IgG1 | PE | Flowcytometry | Abcam | Ab37407 | 100 |

NA: Not available, CST: Cell Signaling Technology, WB: Western blot, ChIP-PCR: Chromatin immunoprecipitation polymerase chain reaction, MACS: Magnetic-activated cell sorting, HRP: horseradish peroxidase.

**Table S3. List of differentially expressed proteins in sox4-overexpressing HCT-116 cells identified by proteomics analysis**

| **Accession** | **Gene Name** | **OE1/C1** | **OE2/C2** | **OE3/C3** |
| --- | --- | --- | --- | --- |
| sp\|O95295\|SNAPN_HUMAN | SNAPIN | 22.7 | 99.08 | 1.6 |
| sp\|Q96EY1\|DNJA3_HUMAN | DNAJA3 | 17.3 | 3.15 | 10.38 |
| sp\|P83881\|RL36A_HUMAN | RPL36A | 6.19 | 1.58 | 2.63 |
| sp\|P26373\|RL13_HUMAN | RPL13 | 6.11 | 1.91 | 1.98 |
| sp\|P48047\|ATPO_HUMAN | ATP5O | 5.75 | 7.31 | 2.91 |
| sp\|P62263\|RS14_HUMAN | RPS14 | 5.7 | 2.3 | 2.33 |
| sp\|O00483\|NDUA4_HUMAN | NDUFA4 | 5.6 | 4.06 | 3.02 |
| sp\|Q9HA64\|KT3K_HUMAN | FN3KRP | 5.37 | 1.53 | 2.29 |
| sp\|P63244\|RACK1_HUMAN | RACK1 | 5.22 | 3.22 | 4.04 |
| sp\|P31350-2\|RIR2_HUMAN | RRM2 | 4.63 | 4.25 | 3.28 |
| sp\|P46013\|KI67_HUMAN | MKI67 | 4.57 | 1.84 | 3.05 |
| sp\|P62854\|RS26_HUMAN | RPS26 | 4.49 | 7.94 | 1.55 |
| sp\|Q13151\|ROA0_HUMAN | HNRNPA0 | 4.21 | 2.37 | 2.24 |
| sp\|Q9BTE6-3\|AASD1_HUMAN | AARSD1 | 4.21 | 2.58 | 1.9 |
| sp\|P63162-2\|RSMN_HUMAN | SNRPN | 4.11 | 8.87 | 3.6 |
| sp\|P62280\|RS11_HUMAN | RPS11 | 4.07 | 2.07 | 5.75 |
| sp\|P31689\|DNJA1_HUMAN | DNAJA1 | 4.06 | 4.72 | 1.59 |
| sp\|P83731\|RL24_HUMAN | RPL24 | 3.73 | 3.27 | 1.73 |
| sp\|P31431\|SDC4_HUMAN | SDC4 | 3.7 | 6.98 | 5.83 |
| sp\|P17844\|DDX5_HUMAN | DDX5 | 3.66 | 5.94 | 2.64 |
| sp\|O00571\|DDX3X_HUMAN | DDX3X | 3.61 | 2.22 | 2.08 |
| sp\|P09234\|RU1C_HUMAN | SNRPC | 3.56 | 6.98 | 5.08 |
| sp\|P61313\|RL15_HUMAN | RPL15 | 3.42 | 1.51 | 1.52 |
| sp\|P63279\|UBC9_HUMAN | UBE2I | 3.37 | 5.27 | 1.83 |
| sp\|P04406\|G3P_HUMAN | GAPDH | 3.36 | 3.7 | 3.19 |
| sp\|Q15388\|TOM20_HUMAN | TOMM20 | 3.25 | 8.32 | 3.4 |
| sp\|P39019\|RS19_HUMAN | RPS19 | 3.24 | 2.75 | 2.43 |
| sp\|P62241\|RS8_HUMAN | RPS8 | 3.16 | 2.25 | 3.33 |
| sp\|P62837\|UB2D2_HUMAN | UBE2D2 | 3.16 | 2.13 | 2.13 |
| sp\|Q9UBQ7\|GRHPR_HUMAN | GRHPR | 3.16 | 1.72 | 1.58 |
| sp\|P62753\|RS6_HUMAN | RPS6 | 3.13 | 1.88 | 2.28 |
| sp\|Q96FW1\|OTUB1_HUMAN | OTUB1 | 3.1 | 2 | 2.3 |
| sp\|Q15942\|ZYX_HUMAN | ZYX | 3.06 | 4.88 | 3.36 |
| sp\|P62851\|RS25_HUMAN | RPS25 | 3.05 | 1.78 | 1.52 |
| sp\|O00148\|DX39A_HUMAN | DDX39A | 3.03 | 1.84 | 3.36 |
| sp\|P27635\|RL10_HUMAN | RPL10 | 3.03 | 1.57 | 1.75 |
| sp\|P62826\|RAN_HUMAN | RAN | 3.02 | 1.93 | 1.64 |
| sp\|Q9NRF8\|PYRG2_HUMAN | CTPS2 | 3.02 | 2.83 | 3.19 |
| sp\|Q01844\|EWS_HUMAN | EWSR1 | 3.01 | 1.94 | 2.54 |
| sp\|P04183\|KITH_HUMAN | TK1 | 2.96 | 4.83 | 2.11 |
| sp\|Q13148\|TADBP_HUMAN | TARDBP | 2.95 | 1.88 | 2.61 |
| sp\|Q86U90\|YRDC_HUMAN | YRDC | 2.91 | 2.56 | 7.94 |
| sp\|P13639\|EF2_HUMAN | EEF2 | 2.9 | 2.06 | 2.16 |
| sp\|P30086\|PEBP1_HUMAN | PEBP1 | 2.82 | 3.36 | 2.45 |
| sp\|Q8TAT6\|NPL4_HUMAN | NPLOC4 | 2.78 | 1.64 | 1.72 |
| sp\|Q9Y2S0\|RPAC2_HUMAN | POLR1D | 2.78 | 1.79 | 1.58 |
| sp\|P62269\|RS18_HUMAN | RPS18 | 2.73 | 1.99 | 2.74 |
| sp\|P84098\|RL19_HUMAN | RPL19 | 2.72 | 2.32 | 2.38 |
| sp\|Q15785\|TOM34_HUMAN | TOMM34 | 2.7 | 1.56 | 1.51 |
| sp\|Q9H845\|ACAD9_HUMAN | ACAD9 | 2.7 | 1.53 | 1.58 |
| sp\|Q9GZL7\|WDR12_HUMAN | WDR12 | 2.56 | 2.07 | 3.56 |
| sp\|P15880\|RS2_HUMAN | RPS2 | 2.55 | 1.91 | 1.53 |
| sp\|Q07020\|RL18_HUMAN | RPL18 | 2.55 | 2.61 | 2.15 |
| sp\|P52292\|IMA1_HUMAN | KPNA2 | 2.52 | 5.25 | 3.6 |
| sp\|O43809\|CPSF5_HUMAN | NUDT21 | 2.52 | 5.5 | 1.7 |
| sp\|Q9H4L7\|SMRCD_HUMAN | SMARCAD1 | 2.51 | 1.72 | 12.94 |
| sp\|P36578\|RL4_HUMAN | RPL4 | 2.51 | 2.03 | 1.84 |
| sp\|Q53F19\|NCBP3_HUMAN | NCBP3 | 2.5 | 2.22 | 3.77 |
| sp\|P23396\|RS3_HUMAN | RPS3 | 2.42 | 1.82 | 1.9 |
| sp\|P49189\|AL9A1_HUMAN | ALDH9A1 | 2.4 | 1.88 | 1.8 |
| sp\|P08238\|HS90B_HUMAN | HSP90AB1 | 2.4 | 2.49 | 2.42 |
| sp\|Q9Y3U8\|RL36_HUMAN | RPL36 | 2.39 | 3.27 | 1.67 |
| sp\|P62750\|RL23A_HUMAN | RPL23A | 2.39 | 2 | 2.3 |
| sp\|Q9NVM6\|DJC17_HUMAN | DNAJC17 | 2.38 | 1.75 | 1.51 |
| sp\|O95249\|GOSR1_HUMAN | GOSR1 | 2.37 | 3.47 | 1.89 |
| sp\|Q71UM5\|RS27L_HUMAN | RPS27L | 2.34 | 3.65 | 1.73 |
| sp\|P62913\|RL11_HUMAN | RPL11 | 2.3 | 2.25 | 2.2 |
| sp\|P07437\|TBB5_HUMAN | TUBB | 2.25 | 3.13 | 2.79 |
| sp\|P08243\|ASNS_HUMAN | ASNS | 2.23 | 2.3 | 2.44 |
| sp\|P18124\|RL7_HUMAN | RPL7 | 2.23 | 2.18 | 2.02 |
| sp\|P11142\|HSP7C_HUMAN | HSPA8 | 2.21 | 2.43 | 1.98 |
| sp\|P62277\|RS13_HUMAN | RPS13 | 2.2 | 1.61 | 1.81 |
| sp\|P23526\|SAHH_HUMAN | AHCY | 2.19 | 1.51 | 1.75 |
| sp\|P23921\|RIR1_HUMAN | RRM1 | 2.18 | 2.42 | 1.87 |
| sp\|Q9P0M9\|RM27_HUMAN | MRPL27 | 2.17 | 5.25 | 3.82 |
| sp\|Q9GZT3\|SLIRP_HUMAN | SLIRP | 2.16 | 2.04 | 2.51 |
| sp\|P68104\|EF1A1_HUMAN | EEF1A1 | 2.16 | 2.29 | 2.39 |
| sp\|Q969X6\|UTP4_HUMAN | UTP4 | 2.15 | 1.67 | 1.66 |
| sp\|P07900-2\|HS90A_HUMAN | HSP90AA1 | 2.13 | 2.72 | 2.29 |
| sp\|P62910\|RL32_HUMAN | RPL32 | 2.13 | 1.7 | 2.4 |
| sp\|P46782\|RS5_HUMAN | RPS5 | 2.12 | 1.94 | 2.05 |
| sp\|P46781\|RS9_HUMAN | RPS9 | 2.12 | 1.98 | 2.22 |
| sp\|P51398\|RT29_HUMAN | DAP3 | 2.12 | 2.86 | 1.85 |
| sp\|P47895\|AL1A3_HUMAN | ALDH1A3 | 2.09 | 2.21 | 2.04 |
| sp\|O00154-4\|BACH_HUMAN | ACOT7 | 2.05 | 1.58 | 2.45 |
| sp\|O00299\|CLIC1_HUMAN | CLIC1 | 2.02 | 1.79 | 2.45 |
| sp\|P07195\|LDHB_HUMAN | LDHB | 2.02 | 1.87 | 2.68 |
| sp\|P21796\|VDAC1_HUMAN | VDAC1 | 2 | 3.91 | 2.07 |
| sp\|P15311\|EZRI_HUMAN | EZR | 2 | 3.16 | 2.3 |
| sp\|Q12834\|CDC20_HUMAN | CDC20 | 1.99 | 2.65 | 1.52 |
| sp\|Q16763\|UBE2S_HUMAN | UBE2S | 1.98 | 2.79 | 2.58 |
| sp\|O00410\|IPO5_HUMAN | IPO5 | 1.94 | 2.14 | 2.05 |
| sp\|Q13347\|EIF3I_HUMAN | EIF3I | 1.93 | 1.95 | 1.58 |
| sp\|Q14258\|TRI25_HUMAN | TRIM25 | 1.92 | 1.85 | 2.12 |
| sp\|P46776\|RL27A_HUMAN | RPL27A | 1.92 | 2.1 | 2.67 |
| sp\|Q14103\|HNRPD_HUMAN | HNRNPD | 1.91 | 2.5 | 2.08 |
| sp\|P22392-2\|NDKB_HUMAN | NME2 | 1.91 | 2.03 | 2.62 |
| sp\|Q08J23\|NSUN2_HUMAN | NSUN2 | 1.88 | 2.67 | 1.52 |
| sp\|O60264\|SMCA5_HUMAN | SMARCA5 | 1.87 | 1.7 | 1.79 |
| sp\|P62316\|SMD2_HUMAN | SNRPD2 | 1.86 | 4.43 | 1.58 |
| sp\|Q13242\|SRSF9_HUMAN | SRSF9 | 1.85 | 2.32 | 2.23 |
| sp\|Q13464\|ROCK1_HUMAN | ROCK1 | 1.84 | 1.56 | 3.77 |
| sp\|P17931\|LEG3_HUMAN | LGALS3 | 1.82 | 2.75 | 1.79 |
| sp\|Q06481-6\|APLP2_HUMAN | APLP2 | 1.81 | 2.36 | 17.7 |
| sp\|Q5JTH9\|RRP12_HUMAN | RRP12 | 1.8 | 1.86 | 1.75 |
| sp\|Q9NXA8-2\|SIR5_HUMAN | SIRT5 | 1.8 | 1.98 | 2.44 |
| sp\|Q9H5V8\|CDCP1_HUMAN | CDCP1 | 1.76 | 1.56 | 1.84 |
| sp\|Q9Y4Z0\|LSM4_HUMAN | LSM4 | 1.75 | 3.3 | 1.58 |
| sp\|Q13283\|G3BP1_HUMAN | G3BP1 | 1.74 | 1.65 | 2.29 |
| sp\|O00541\|PESC_HUMAN | PES1 | 1.71 | 1.97 | 3.05 |
| sp\|Q14247\|SRC8_HUMAN | CTTN | 1.7 | 3.56 | 2.25 |
| sp\|Q9P258\|RCC2_HUMAN | RCC2 | 1.67 | 2 | 2.43 |
| sp\|P01008\|ANT3_HUMAN | SERPINC1 | 1.67 | 4.33 | 2.08 |
| sp\|Q92804\|RBP56_HUMAN | TAF15 | 1.66 | 2.13 | 1.87 |
| sp\|Q7L2H7\|EIF3M_HUMAN | EIF3M | 1.66 | 1.85 | 1.64 |
| sp\|P60866\|RS20_HUMAN | RPS20 | 1.66 | 3.5 | 1.95 |
| sp\|P46783\|RS10_HUMAN | RPS10 | 1.64 | 2.54 | 2.07 |
| sp\|Q05639\|EF1A2_HUMAN | EEF1A2 | 1.62 | 2.09 | 1.98 |
| sp\|Q96RS6\|NUDC1_HUMAN | NUDCD1 | 1.6 | 1.77 | 3.22 |
| sp\|Q86V81\|THOC4_HUMAN | ALYREF | 1.6 | 2.81 | 1.82 |
| sp\|P42704\|LPPRC_HUMAN | LRPPRC | 1.6 | 1.62 | 1.53 |
| sp\|P36551\|HEM6_HUMAN | CPOX | 1.6 | 3.61 | 2.21 |
| sp\|P09001\|RM03_HUMAN | MRPL3 | 1.59 | 1.92 | 1.8 |
| sp\|P68371\|TBB4B_HUMAN | TUBB4B | 1.58 | 3.19 | 2.27 |
| sp\|Q13547\|HDAC1_HUMAN | HDAC1 | 1.57 | 1.92 | 1.66 |
| sp\|Q6DKK2\|TTC19_HUMAN | TTC19 | 1.55 | 1.51 | 2.33 |
| sp\|P07384\|CAN1_HUMAN | CAPN1 | 1.54 | 1.61 | 1.76 |
| sp\|P62888\|RL30_HUMAN | RPL30 | 1.53 | 1.94 | 1.55 |
| sp\|Q8TD22\|SFXN5_HUMAN | SFXN5 | 0.01 | 0.01 | 0.01 |
| sp\|P80303\|NUCB2_HUMAN | NUCB2 | 0.02 | 0.05 | 0.13 |
| sp\|Q9BRP4\|PAAF1_HUMAN | PAAF1 | 0.04 | 0.43 | 0.48 |
| sp\|Q9H0P0\|5NT3A_HUMAN | NT5C3A | 0.04 | 0.03 | 0.29 |
| sp\|P26232-5\|CTNA2_HUMAN | CTNNA2 | 0.04 | 0.07 | 0.16 |
| sp\|Q9NRY4\|RHG35_HUMAN | ARHGAP35 | 0.05 | 0.01 | 0.06 |
| sp\|Q9HCE1\|MOV10_HUMAN | MOV10 | 0.05 | 0.5 | 0.55 |
| sp\|P55212\|CASP6_HUMAN | CASP6 | 0.05 | 0.2 | 0.13 |
| sp\|Q96NF6\|CH049_HUMAN | C8orf49 | 0.06 | 0.02 | 0.13 |
| sp\|P16104\|H2AX_HUMAN | H2AFX | 0.07 | 0.16 | 0.21 |
| sp\|Q99442\|SEC62_HUMAN | SEC62 | 0.07 | 0.11 | 0.02 |
| sp\|O00754\|MA2B1_HUMAN | MAN2B1 | 0.09 | 0.2 | 0.39 |
| sp\|Q969S9-2\|RRF2M_HUMAN | GFM2 | 0.09 | 0.14 | 0.09 |
| sp\|O43708\|MAAI_HUMAN | GSTZ1 | 0.1 | 0.06 | 0.12 |
| sp\|Q6PI78\|TMM65_HUMAN | TMEM65 | 0.1 | 0.52 | 0.39 |
| sp\|Q9P246-2\|STIM2_HUMAN | STIM2 | 0.11 | 0.28 | 0.63 |
| sp\|Q14789\|GOGB1_HUMAN | GOLGB1 | 0.11 | 0.41 | 0.58 |
| sp\|Q6ZVM7\|TM1L2_HUMAN | TOM1L2 | 0.12 | 0.01 | 0.42 |
| sp\|Q8N8S7-3\|ENAH_HUMAN | ENAH | 0.13 | 0.18 | 0.14 |
| sp\|Q9BR76\|COR1B_HUMAN | CORO1B | 0.13 | 0.57 | 0.28 |
| sp\|P62805\|H4_HUMAN | HIST1H4A | 0.14 | 0.06 | 0.13 |
| sp\|Q494V2\|CP100_HUMAN | CFAP100 | 0.15 | 0.18 | 0.1 |
| sp\|Q9BQ67\|GRWD1_HUMAN | GRWD1 | 0.15 | 0.62 | 0.61 |
| sp\|Q03135\|CAV1_HUMAN | CAV1 | 0.16 | 0.43 | 0.24 |
| sp\|Q8TCS8\|PNPT1_HUMAN | PNPT1 | 0.17 | 0.44 | 0.58 |
| sp\|P68431\|H31_HUMAN | HIST1H3A | 0.18 | 0.06 | 0.14 |
| sp\|P61916\|NPC2_HUMAN | NPC2 | 0.19 | 0.26 | 0.35 |
| sp\|P55145\|MANF_HUMAN | MANF | 0.2 | 0.42 | 0.43 |
| sp\|O94808\|GFPT2_HUMAN | GFPT2 | 0.21 | 0.25 | 0.48 |
| sp\|Q5T6F2\|UBAP2_HUMAN | UBAP2 | 0.22 | 0.35 | 0.4 |
| sp\|Q9H4G0\|E41L1_HUMAN | EPB41L1 | 0.22 | 0.6 | 0.29 |
| sp\|P57075\|UBS3A_HUMAN | UBASH3A | 0.24 | 0.04 | 0.13 |
| sp\|P00568\|KAD1_HUMAN | AK1 | 0.25 | 0.6 | 0.51 |
| sp\|Q92597\|NDRG1_HUMAN | NDRG1 | 0.27 | 0.21 | 0.3 |
| sp\|O94992\|HEXI1_HUMAN | HEXIM1 | 0.27 | 0.31 | 0.43 |
| sp\|Q99880\|H2B1L_HUMAN | HIST1H2BL | 0.28 | 0.15 | 0.05 |
| sp\|P04004\|VTNC_HUMAN | VTN | 0.28 | 0.17 | 0.37 |
| sp\|O75367\|H2AY_HUMAN | H2AFY | 0.3 | 0.18 | 0.39 |
| sp\|Q9BPX3\|CND3_HUMAN | NCAPG | 0.31 | 0.15 | 0.26 |
| sp\|P09972\|ALDOC_HUMAN | ALDOC | 0.32 | 0.33 | 0.37 |
| sp\|Q96B23-2\|CR025_HUMAN | C18orf25 | 0.32 | 0.01 | 0.3 |
| sp\|P62979\|RS27A_HUMAN | RPS27A | 0.32 | 0.48 | 0.38 |
| sp\|Q9Y5U9\|IR3IP_HUMAN | IER3IP1 | 0.33 | 0.6 | 0.39 |
| sp\|P35240\|MERL_HUMAN | NF2 | 0.33 | 0.43 | 0.51 |
| sp\|Q9BWD1\|THIC_HUMAN | ACAT2 | 0.34 | 0.55 | 0.63 |
| sp\|P07910-2\|HNRPC_HUMAN | HNRNPC | 0.35 | 0.38 | 0.24 |
| sp\|P02765\|FETUA_HUMAN | AHSG | 0.35 | 0.32 | 0.17 |
| sp\|Q8NHP6\|MSPD2_HUMAN | MOSPD2 | 0.36 | 0.32 | 0.36 |
| sp\|P48960\|CD97_HUMAN | CD97 | 0.37 | 0.13 | 0.24 |
| sp\|Q969M3-3\|YIPF5_HUMAN | YIPF5 | 0.37 | 0.26 | 0.54 |
| sp\|P20020\|AT2B1_HUMAN | ATP2B1 | 0.38 | 0.33 | 0.22 |
| sp\|Q8N5K1\|CISD2_HUMAN | CISD2 | 0.39 | 0.62 | 0.3 |
| sp\|Q14353\|GAMT_HUMAN | GAMT | 0.39 | 0.51 | 0.29 |
| sp\|P01023\|A2MG_HUMAN | A2M | 0.4 | 0.22 | 0.4 |
| sp\|Q96T23\|RSF1_HUMAN | RSF1 | 0.4 | 0.51 | 0.31 |
| sp\|P13674\|P4HA1_HUMAN | P4HA1 | 0.41 | 0.34 | 0.28 |
| sp\|P02788\|TRFL_HUMAN | LTF | 0.42 | 0.37 | 0.4 |
| sp\|Q14139\|UBE4A_HUMAN | UBE4A | 0.42 | 0.39 | 0.52 |
| sp\|Q8N3R9\|MPP5_HUMAN | MPP5 | 0.44 | 0.24 | 0.08 |
| sp\|Q8NEJ9\|NGDN_HUMAN | NGDN | 0.44 | 0.65 | 0.28 |
| sp\|Q9H4M9\|EHD1_HUMAN | EHD1 | 0.45 | 0.52 | 0.58 |
| sp\|Q14126\|DSG2_HUMAN | DSG2 | 0.45 | 0.2 | 0.42 |
| sp\|Q6GMV2\|SMYD5_HUMAN | SMYD5 | 0.47 | 0.48 | 0.45 |
| sp\|Q13425\|SNTB2_HUMAN | SNTB2 | 0.47 | 0.07 | 0.19 |
| sp\|Q8WXA2\|PATE1_HUMAN | PATE1 | 0.48 | 0.21 | 0.3 |
| sp\|P02768\|ALBU_HUMAN | ALB | 0.48 | 0.25 | 0.43 |
| sp\|Q9H8Y5\|ANKZ1_HUMAN | ANKZF1 | 0.49 | 0.01 | 0.26 |
| sp\|Q9NZB2-6\|F120A_HUMAN | FAM120A | 0.49 | 0.59 | 0.34 |
| sp\|Q9P275\|UBP36_HUMAN | USP36 | 0.51 | 0.51 | 0.66 |
| sp\|P02647\|APOA1_HUMAN | APOA1 | 0.52 | 0.35 | 0.46 |
| sp\|P18085\|ARF4_HUMAN | ARF4 | 0.53 | 0.64 | 0.56 |
| sp\|O14908\|GIPC1_HUMAN | GIPC1 | 0.53 | 0.59 | 0.47 |
| sp\|O95573\|ACSL3_HUMAN | ACSL3 | 0.55 | 0.63 | 0.52 |
| sp\|Q5TFE4\|NT5D1_HUMAN | NT5DC1 | 0.55 | 0.57 | 0.6 |
| sp\|Q15392\|DHC24_HUMAN | DHCR24 | 0.55 | 0.52 | 0.47 |
| sp\|Q8IUC4\|RHPN2_HUMAN | RHPN2 | 0.56 | 0.43 | 0.21 |
| sp\|O15479\|MAGB2_HUMAN | MAGEB2 | 0.57 | 0.39 | 0.5 |
| sp\|Q66K14\|TBC9B_HUMAN | TBC1D9B | 0.58 | 0.35 | 0.43 |
| sp\|Q15154\|PCM1_HUMAN | PCM1 | 0.58 | 0.35 | 0.63 |
| sp\|Q15057\|ACAP2_HUMAN | ACAP2 | 0.58 | 0.4 | 0.45 |
| sp\|Q13011\|ECH1_HUMAN | ECH1 | 0.59 | 0.31 | 0.16 |
| sp\|Q9P015\|RM15_HUMAN | MRPL15 | 0.6 | 0.59 | 0.04 |
| sp\|Q9BZJ0\|CRNL1_HUMAN | CRNKL1 | 0.61 | 0.61 | 0.08 |
| sp\|P32929\|CGL_HUMAN | CTH | 0.64 | 0.17 | 0.63 |
| sp\|Q96EK5\|KBP_HUMAN | KIF1BP | 0.65 | 0.6 | 0.28 |
| sp\|O94901-9\|SUN1_HUMAN | SUN1 | 0.66 | 0.65 | 0.51 |
| sp\|Q92542\|NICA_HUMAN | NCSTN | 0.66 | 0.51 | 0.55 |

OE: SOX4-overexpressing cells. C: Control cells with empty plasmid transfection

**Table S4. Top 5 PPI network modules as identified using MCODE in Cytoscape. Module score > 5 is considered as significant. HDAC1 is involved in Module 4.**

| **Module #** | **Module Score** | **Major Pathways** |
| --- | --- | --- |
| **1** | 39.641 | IL2 signaling events mediated by PI3K and Activation of the mRNA upon binding of the cap-binding complex and eIFs, and subsequent binding to 43S. |
| **2** | 10.727 | Gene Expression and mRNA Splicing |
| **3** | 6.000 | DNA Double Strand Break Response and Cell cycle_Spindle assembly and chromosome separation. |
| **4** | 5.667 | Signaling by Wnt and Activated PKN1 stimulates transcription of AR (androgen receptor) regulated genes KLK2 and KLK3 |
| **5** | 5.200 | Lipoprotein metabolism and Binding and Uptake of Ligands by Scavenger Receptors |
